# Quantitative assessment of extravasation of IL-15-secreting MSLN-CAR-NK-92 cells using tumor transparency imaging

**DOI:** 10.7150/thno.125194

**Published:** 2026-04-23

**Authors:** Sera Hong, Dohyeon Moon, Seoin Hwang, Mijeong Lee, Duck Cho, Joon Myong Song

**Affiliations:** 1 College of Pharmacy, Seoul National University, Seoul 08826, Republic of Korea.; 2 Department of Health Sciences and Technology, Samsung Advanced Institute for Health Sciences and Technology, Sungkyunkwan University, Seoul 06351, Republic of Korea.; 3 Department of Laboratory Medicine and Genetics, Samsung Medical Center, Sungkyunkwan University School of Medicine, Seoul 06351, Republic of Korea.

**Keywords:** CAR-NK cell, extravasation, drug permeability, pancreatic cancer, tumor transparent imaging

## Abstract

**Background:**

The extravasation of anticancer immune cells is a very important issue that must be understood to improve the anticancer effect of chimeric antigen receptor (CAR)-expressing anticancer immune cell therapy. To date, no study has been reported to quantitatively evaluate the degree of extravasation of anticancer immune cells escaping from tumor blood vessels to the tumor microenvironment (TME) at the microscopic level.

**Methods:**

In this study, for the first time, using tumor transparency imaging, the extent of extravasation of CAR-NK and NK cells in pancreatic tumors was determined. we used tumor transparency imaging, which preserves intact vasculature, to accurately measure the extravasation and infiltration of established MSLN-CAR-NK-92 cells and unmodified NK-92 cells in an NSG mouse model of pancreatic cancer. Extravasation was quantified by calculating the volume ratio of cells located inside versus outside tumor vessels.

**Results:**

Following intravenous infusion, MSLN-CAR-NK-92 cells showed higher extravasation rates (85.3% vs. 57.4%), penetration depths (185 μm vs. 128 μm), and average extravasated cell counts (7,717 vs. 2,311) compared with NK-92 cells. Further measures of penetration and cytotoxicity also favored MSLN-CAR-NK-92 cells, with CPA50/CPD50 values of 6,887 cells at 88 μm versus 3,509 cells at 45 μm, and CDA50/CDD50 values of 6,350 cells at 102 μm versus 2,023 cells at 48 μm, respectively. These findings highlight the value of extravasation efficiency as a metric for assessing immune cell performance in solid tumors.

**Conclusion:**

Considering these results, the extravasation efficiency of anticancer immune cells can be regarded as a valuable indicator for evaluating the effectiveness of CAR constructs designed for NK cells target pancreatic cancer. In this study, we establish a quantitative extravasation imaging platform for evaluating CAR-NK cell trafficking in pancreatic and cholangiocarcinoma tumor models. This approach provides a structured framework for assessing immune cell delivery and therapeutic distribution within the tumor microenvironment.

## Introduction

Chimeric antigen receptor-engineered natural killer (CAR-NK) cells have demonstrated strong antitumor activity, particularly against hematological malignancies. However, in solid tumors such as pancreatic ductal adenocarcinoma (PDAC), dense stroma, abnormal vasculature, and immunosuppressive cytokines hinder immune cell infiltration and persistence, thereby limiting therapeutic efficacy 1, 2. Mesothelin is a tumor-associated antigen highly expressed in PDAC and several other malignancies 3-5, making it a promising immunotherapeutic target. IL-15 secretion enhances NK cell proliferation, survival, and antitumor activity 6. Although mesothelin-targeting, IL-15-secreting CAR-NK cells have been extensively studied for their cytotoxic potency 7, their *in vivo* trafficking behavior within the intact tumor microenvironment (TME) remains insufficiently characterized. In particular, quantitative evaluation of immune cell extravasation, the process by which immune cells exit tumor vasculature into the surrounding tissue, has been lacking despite its relevance as a functional indicator of antitumor efficacy 8, 9. Conventional histological methods rely on thin tissue sections that disrupt three-dimensional (3D) architecture and vascular continuity, limiting accurate spatial assessment of immune cell infiltration. To address this limitation, we employed, for the first time, a tissue optical clearing technology called CLARITY-based tissue optical clearing for tumor transparency imaging. This method removes lipid bilayers while preserving proteins and nucleic acids, producing an optically transparent nanoporous hydrogel-tissue hybrid that enables high-resolution, volumetric 3D imaging of labeled immune cells within unsectioned tumors while maintaining their spatial relationships with the tumor vasculature 10-13. In this study, MSLN-CAR-NK-92 cells and IL-15-secreting MSLN-CAR-NK-92 cells were intravenously administered into a PANC-1 PDAC xenograft mouse model, and used tumor transparency imaging to quantitatively assess their extravasation efficiency, penetration depth, and spatial distribution relative to blood vessels, in comparison with unmodified NK-92 cells. We present a quantitative comparative analysis of the extravasation efficiency of MSLN-CAR-NK-92 and NK-92 cells as they exit tumor blood vessels. Extravasation has gained considerable attention as a key indicator of the anticancer efficacy of immune cells within tumors 14, 15. Numerous studies have investigated the mechanisms underlying immune cell extravasation, as increased extravasation correlates with enhanced ability of anticancer immune cells to eliminate tumor cells 15-17. Our findings provide the first quantitative 3D analysis of CAR NK cell extravasation in PDAC, offering a robust framework for evaluating immune cell trafficking and infiltration in solid tumors. This supports the application of advanced imaging methods for accurate biodistribution studies in cell-based immunotherapy.

## Materials and Methods

### Cell lines

K562 (CCL-243) and PANC-1 (CRL-1469) cells were obtained from American Type Culture Collection (ATCC, Manassas, VA, USA), Huh-7 cells (KCLB No. 60104**)** were obtained from Korea Cell Line Bank (KLCB, Seoul, Republic of Korea) and HuCCT1 cells (RCB1960) were obtained from RIKEN BRC Cell Bank (Tsukuba, Japan). The coding sequence of human mesothelin was amplified by PCR from cDNA synthesized using total RNA extracted from PANC-1 cells, and the PCR product was subsequently cloned into the pCDH-MSCV vector (System Biosciences, Palo Alto, CA, USA). An mesothelin expression plasmid was used to generate mesothelin-overexpressing K562 cells. Firefly luciferase-expressing viral particles were purchased from Lugen Sciences (Shanghai, China). Co., Ltd. (Bucheon, Gyunggi-do, Republic of Korea) and used to establish luciferase-overexpressing Huh-7 and PANC-1 cell lines.

### Cell culture

K562 was cultured with RPMI 1640 medium (Cytiva, Logan, UT, USA), completed with 10% heat-inactivated FBS (Thermo Fisher Scientific, Waltham, MA, USA), 100 U/mL penicillin, 100 μg/mL streptomycin (15140122, Gibco, Grand Island, NY, USA). Huh-7, PANC-1 and Lenti-X 293T cells were cultured with DMEM medium (Gibco, Grand Island, NY, USA) completed with 10% heat-inactivated FBS, 100 U/mL penicillin, 100 ug/mL streptomycin. NK-92 was cultured with 200 U/mL of IL-2 (PeproTech, Rocky Hill, NJ, USA) added MEM-α medium (Gibco, Grand Island, NY, USA) completed with 10% heat-inactivated FBS, 10% heat-inactivated Horse Serum (Gibco, Grand Island, NY, USA), 100 U/mL penicillin, 100 μg/mL streptomycin, 0.2 mM Myo-inositol (Sigma-Aldrich, St. Louis, MO, USA), 0.02 mM Folic Acid (Sigma-Aldrich, St. Louis, MO, USA), 0.1 mM 2-Mercaptoethanol (Gibco, Grand Island, NY, USA). The PANC-1 human pancreatic cancer cells were cultured as monolayers in Dulbecco's Modified Eagle's Medium (Cat. No. LM 001-05; Welgene, Gyeongsan, Gyeongsangbuk-do, Republic of Korea) supplemented with 10% heat-inactivated FBS (Cat. No. 16000044; Gibco, Grand Island, NY, USA) and 1% antibiotic-antimycotic solution (Cat. No. 15240062; Gibco, Grand Island, NY, USA) at 37 °C in a 5% CO_2_ incubator. The HuCCT1 human bile duct carcinoma cell line was maintained in RPMI 1640 medium (Cat. No. 11875093; Gibco, Grand Island, NY, USA) supplemented with 10% fetal bovine serum, 1% penicillin-streptomycin at 37 °C in a humidified incubator with a 5% CO_2_.

### CAR construct design / plasmid construction

All MSLN (SS1 clone)-targeted CAR constructs used in this study were second-generation CARs. To enable IL-15 co-expression, a synthetic IL-15 sequence was constructed by linking the CD33 signal peptide (NM_001082618.2) to the mature IL-15 region (NM_000585.5) according to previously described methods 18. Two constructs were generated: one encoding only the MSLN-specific CAR, and the other co-expressing IL-15 via a T2A peptide. Gene synthesis was performed by Lugen Sci Co., Ltd. (Bucheon, Gyeonggi-do, Republic of Korea), and constructs were assembled using the In-Fusion cloning technique (Takara Bio, Shiga, Japan).

### Lentivirus production & transduction

Lentiviral particles were produced by co-transfecting the Lenti-X 293T cell line with the transfer plasmid and packaging plasmids psPAX2 and pMD2.G using Lipofectamine 2000 (Thermo Fisher Scientific, Waltham, MA, USA), following the manufacturer's protocol. Viral supernatants were harvested 72 h post-transfection, filtered through a 0.45 µm PVDF filter (Advantec, Tokyo, Japan), and concentrated. For concentration, Lenti-Con Reagent (Lugen Sci, Co., Ltd., Bucheon, Gyeonggi-do, Republic of Korea) was added to the filtered supernatant and incubated at 4 °C for 16 h. The mixture was then centrifuged at 1,600 × g for 50 min at 4 °C, and the viral particles were resuspended in DMEM and stored at -80 °C. For transduction, NK-92 cells were seeded in retronectin-coated 24-well plates (Takara Bio) and incubated with concentrated lentiviral particles. Plates were spinoculated at 1,000 × g for 90 min at 32 °C and then incubated at 37 °C. Transduction efficiency was assessed by flow cytometry 72 h post-transduction.

### Flow cytometry-based cytotoxicity assay

For short-term cytotoxicity assays, K562 or K562-mesothelin-OE (overexpression) target cells were labeled with V450 dye (Invitrogen™, Oregon, USA) and co-cultured with effector NK-92 cells—NK-92, IL-15 OE NK-92, MSLN-CAR-NK-92, or MSLN-CAR-IL-15 OE NK-92—at the indicated E:T ratios in round-bottom 96-well plates (SPL, Pocheon-si, Gyeonggi-do, Republic of Korea). A total of 1 × 10^4^ target cells were used per well, and co-culture was performed at 37 °C for 4 h. After incubation, cells were stained with propidium iodide (Invitrogen™, Waltham, MA, USA) to assess target cell cytotoxicity. For long-term cytotoxicity assays, APC-labeled K562 or K562-mesothelin-OE target cells were co-cultured with the same panel of effector NK-92 cells in round-bottomed 96-well plates at an E:T ratio of 1:4. In total, 1-10 target cells were seeded per well. At 24, 48, and 72 h, the cells were harvested and stained with anti-CD56-PE-Cy7 (eBioscience, San Diego, CA, USA) to distinguish NK-92 cells, along with a viability dye to evaluate target cell survival. The percentages of target cells were measured at each time point.

### Luciferase-based cytotoxicity assay

For adherent target cells, Huh-7-luc or PANC-1-luc cells were seeded at 5 × 10^4^ cells per well in 96-well white plates (SPL, Pocheon-si, Gyeonggi-do, Republic of Korea) one day prior to co-culture. The following day, NK-92 effector cells—NK-92, MSLN-CAR-NK-92, and MSLN-CAR-IL-15-NK-92 cells—were added at indicated E:T ratios. Co-culture was carried out at 37 °C for the indicated durations. Immediately prior to luminescence measurement, D-luciferin (PerkinElmer, Boston, MA, USA) was added to each well and luminescence was measured using a microplate luminometer (Mithras LB 943, Berthold Technologies GmbH & Co., Wildbad, Germany). Relative luminescence units (RLU) were used to assess the viability of the luciferase-expressing target cells, and decreased RLU values indicated increased cytotoxicity.

### Cytokine measurement

NK and target cells were co-cultured in duplicates at an E:T ratio of 1:1, with 2 × 10^5^ cells of each type seeded in 96-well round-bottom plates. After 24 h, the cells were centrifuged and the supernatant was collected for cytokine measurements. Human IFN-γ and IL-15 levels were quantified using ELISA kits (BD Biosciences, Franklin Lakes, NJ, USA) according to the manufacturer's instructions. Absorbance was measured at 450 nm using a microplate reader (SpectraMax ABS Plus; Molecular Devices, Shanghai, China), and cytokine concentrations were calculated based on standard curves generated using known concentrations of recombinant cytokines.

### Degranulation assay

NK-92 cells (2 × 10^4^) were co-cultured with target cells (K562, K562-mesothelin-OE, Huh-7, and PANC-1) at an E:T ratio of 1:2 in round-bottom 96-well plates at 37 °C for 4 h. During co-culture, anti-CD107a-PE antibody (BD Pharmingen™, San Diego, CA, USA) and monensin (Invitrogen™, Waltham, MA, USA) were added to block cytokine and granule secretion. After incubation, the cells were washed and analyzed by flow cytometry.

### Cell survival/proliferation assay

NK-92 cells were seeded at a density of 3 × 10^4^ cells per well in 96-well flat-bottom plates and cultured for up to 7 days. Cell viability was assessed on days 0, 4, and 7 using propidium iodide (PI) staining, and only viable cells were counted. Absolute cell numbers were quantified by flow cytometry using CountBright™ Absolute Counting Beads (Invitrogen™, Waltham, MA, USA).

### Pancreatic xenograft models

Subcutaneous PANC-1 pancreatic cancer xenografts were established by inoculating NOD-SCID IL2Rγ^null^ (NSG) mice with 1 × 10^6^ PANC-1 cells suspended in 100 μL of PBS mixed with 100 μL of Geltrex™, injected into the right flank. Tumor growth was measured using a caliper, and tumor volumes were calculated using the following formula: length × (width)^2^ × 0.5. For each experiment, a total of five NSG mice were used (biological replicates, n = 5). When tumor volumes reached approximately 1,000 mm^3^, the animals were treated via intravenous injection of 1 × 10^7^ NK-92 (GFP-expressing or CellTrace™-labeled) and/or MSLN-CAR-NK-92 (GFP- or IL-15 expressing) cells. The control mice were injected of PBS. 48 h after injection, to visualize blood vessels, mice were intravenously injected with 100 μg of *Lycopersicon Esculentum* (Tomato) Lectin-DyLight® 649 in 100 μL of PBS (Cat. No. DL-1178-1; Vector Laboratories, Newark, CA, USA; excitation/emission: 655/670 nm). After 20 min, mice were euthanized via CO_2_ inhalation and tumors were immediately excised. The harvested tumors were fixed in 4% paraformaldehyde (PFA) for 24 h at 4 °C, washed with 1× PBS, and stored in 1× PBS at 4 °C until further experiments.

### Cholangiocarcinoma xenograft models

Subcutaneous HuCCT1 cholangiocarcinoma xenografts were established by inoculating NOD-SCID IL2Rγ^null^ (NSG) mice with 1 × 10^6^ HuCCT1 cells suspended in 100 μL of PBS mixed with 100 μL of Geltrex™, injected into the right flank (biological replicates, n = 5). When tumor volumes reached approximately 1,000 mm^3^, the animals were treated via intravenous injection of 1 × 10^7^ MSLN-CAR-NK-92-GFP cells. 48 h after injection, to visualize blood vessels, mice were intravenously injected with 100 μg of *Lycopersicon Esculentum* (Tomato) Lectin-DyLight® 649 in 100 μL of PBS (Cat. No. DL-1178-1; Vector Laboratories, Newark, CA, USA; excitation/emission: 655/670 nm). After 20 min, mice were euthanized via CO_2_ inhalation and tumors were immediately excised. The harvested tumors were fixed in 4% paraformaldehyde (PFA) for 24 h at 4 °C, washed with 1× PBS, and stored in 1× PBS at 4 °C until further experiments.

### Tissue clearing

Tumors fixed in PFA were sectioned into 1 mm slices and immersed in X-CLARITY™ hydrogel solution supplemented with Polymerization Initiator (Cat. No. C1310X; Logos Biosystems, Anyang, Gyeonggi-do, Republic of Korea) at 4 °C for 24 h. Nitrogen gas was applied for 3 min to remove oxygen from the sample chamber. The tissues were then polymerized by placing the container in a 37 °C water bath for 3 h. Subsequently, X-CLARITY tissue-clearing system (Cat. No. C30001; Logos Biosystems, Anyang, Gyeonggi-do, Republic of Korea) was used for active clearing, operating at 1.0 A and 37 °C. The system employed 1.2 L of electrophoretic tissue-clearing solution (Cat. No. C13001; Logos Biosystems, Anyang, Gyeonggi-do, Republic of Korea), enabling efficient lipid removal from the tissues through continuous solution circulation. This clearing solution, formulated with 4% SDS at pH 8.5, is integral to the process. After clearing, the tissues were washed with 1× PBS at 37 °C.

### Immunostaining for apoptotic cell death detection

To identify NK-92 and MSLN-CAR-NK-92 cells-induced apoptotic cancer cells, clear tumor tissues with a thickness of 1 mm were stained using an *in situ* Apoptosis Detection Kit (Cat. No. MK500; Takara Bio, Shiga, Japan). Initially, the tissues were treated with permeabilization buffer at 4 °C for 4 h. Then, they were incubated overnight at 37 °C in a tabletop incubator with a staining solution containing TdT enzyme, Hoechst 33342, and fluorescein-dUTP containing labeling-safe buffer. Afterward, the tissues were washed with 1× PBS at 37 °C. Finally, the stained tissues were rinsed three times with double-distilled water (DDW) and immersed in X-CLARITY™ mounting solution (refractive index = 1.46; Cat, No. C13101; Logos Biosystems, Anyang, Gyeonggi-do, Republic of Korea) at 25 °C before confocal imaging.

### Immunostaining for mesothelin detection

To detect mesothelin-expressing cancer cells in pancreatic PANC-1 tumors, 1-mm-thick cleared tumor tissues were stained with Alexa Fluor® 594-conjugated anti-mesothelin antibody (Cat. No. sc-166124 AF594; Santa Cruz Biotechnology, Dallas, TX, USA) diluted 1:100 in a labeling solution containing 6% (v/v) BSA, 0.2% (v/v) Triton X-100, 0.01% (v/v) sodium azide, and 1× PBS. Following staining, the tissues were washed with 0.2% PBST and incubated with Hoechst 33342 solution. The samples were rinsed with 0.2% PBST in a shaking chamber. Finally, the stained tissues were washed three times with double-distilled water (DDW) and immersed in X-CLARITY™ mounting solution (RI = 1.46, Logos Biosystems) at room temperature prior to confocal imaging.

### 3D transparent imaging in pancreatic tumor

To visualize the distribution of NK-92 / MSLN-CAR-NK-92 cells or apoptotic cell death (TUNEL) in the pancreatic tissue, the cleared tumor samples were placed in a confocal dish between a cover glass and dish base after incubation in the mounting solution. Imaging was conducted using a Leica TCS SP8 DMI8-CS system fitted with a HC PL APO CS 10×/0.40 DRY objective lens (Leica Microsystems GmbH, Wetzlar, Germany). 3D fluorescence images were captured using the Z-stacking function of the system, with the excitation/emission wavelengths set as follows: Hoechst 33342 at 405 nm/435-465 nm, NK-92-GFP cells / MSLN-CAR-NK-92-GFP cells or TUNEL at 488 nm/510-530 nm, NK-CTY or mesothelin at 561 nm/595-620 nm, and lectin-labeled blood vessels at 633 nm/665-685 nm. 3D Z-stack images were acquired across a depth of approximately 300 µm with a z-step interval of 8 µm using sequential scanning to avoid spectral overlap. Images were captured at a resolution of 1024 × 1024 pixels. Three independent subcutaneous PANC-1 tumor-bearing mice were used for imaging (biological replicates, n = 3). For each mouse, three distinct tumor regions were imaged, resulting in a total of nine regions. For quantitative analysis and statistical evaluation, one representative image per mouse was selected based on consistency with the overall pattern observed across the multiple regions, yielding n = 3 biologically independent samples.

### 3D image analysis for distribution of NK-92 / MSLN-CAR-NK-92 cells / apoptotic cell deaths in pancreatic tumor

Three-dimensional (3D) z-stack fluorescence datasets were reconstructed and analyzed using Imaris CL and XT software (version 10.2.0; Bitplane AG, Zürich, Switzerland). All z-stack images were first rendered into volumetric datasets, and fluorescence signals were segmented using intensity-based surface reconstruction. First, nuclear spots were detected using the “Spots” module. Channel 1 (blue, Hoechst-nucleus) was selected as the source channel. The estimated XY diameter was set to 5 μm based on measurements obtained using the measurement point function in slice view. The “Quality” filter was applied, and only the lower threshold was manually adjusted to values above 10 to exclude background noise. Second, blood vessels were segmented using the “Surface” module based on intensity-driven 3D reconstruction. Channel 3 (red, lectin-labeled vessels) was selected as the source channel, and the entire image was designated as the region of interest (ROI). Surface smoothing was applied (surface detail: 2.0 μm) to reduce high-frequency noise while preserving vascular morphology. Background subtraction with an optimized sphere diameter was applied to enhance vessel contrast. Threshold values were determined using the lower-threshold control to accurately reconstruct continuous three-dimensional vascular surfaces and were applied consistently within each experimental group. Third, NK-92-GFP / MSLN-CAR-NK-92-GFP cells or TUNEL-positive apoptotic signals were segmented using the “Surface” module from channel 2 (green, GFP or fluorescein signal). The entire image was set as ROI. Surface smoothing (surface detail: 2.0 μm) and background subtraction were applied, followed by absolute intensity thresholding to reconstruct discrete three-dimensional cellular surfaces. To identify nuclei associated with NK cells or apoptotic cells, the “Find spots close to surface” function was performed using reconstructed NK or TUNEL surfaces as reference objects. A proximity threshold of 5 μm was applied to define nuclei spatially associated with GFP-positive or apoptotic surfaces. For spatial quantification, a 3D distance transformation was performed using the reconstructed blood vessel surfaces. The distance transform mode was set to “outside surface object,” enabling calculation of the minimum Euclidean distance between each nucleus-associated NK or apoptotic cell and the nearest vascular surface. Distance values were extracted from the generated distance transformation channel and used for quantitative analysis. Finally, 3D-reconstructed images were generated using surface-rendered objects for blood vessels, NK cells, apoptotic cells, and nuclei. These rendered datasets were used to visualize and quantify the spatial relationship of immune cells, tumor vasculature, and apoptotic tumor cells within the tumor microenvironment.

### 3D image analysis for evaluating extravasation of NK-92 and MSLN-CAR-NK-92 cells in pancreatic tumor

The 3D fluorescence images were analyzed using Imaris CL and XT software (version 10.2.0; Bitplane AG, Zürich, Switzerland) to quantitatively assess the degree of extravasation of NK-92 (CellTrace™-labeled) and MSLN-CAR-NK-92-GFP cells. First, the 'surface' tool was used to segment blood vessels, utilizing channel 4 (red, representing blood vessels) as the source channel. The entire image was designated as the ROI and segmentation was achieved by applying an optimal smoothing filter and setting a threshold. Next, the segmented blood vessels were selected, and the 'Mask All' function in the 'Edit' tool was used to separate the fluorescence signals of intravascular and extravasated NK cells. For intravascular NK cells, voxel intensities outside the vessel surface were set to zero, whereas for extravasated NK cells, voxel intensities inside the vessel surface were set to zero. This process utilized channels 2 (green, representing MSLN-CAR-NK-92 cells) and 3 (yellow, representing NK-92 cells). Finally, the 'Surface' tool was applied to segment intravascular and extravasated NK-92 and MSLN-CAR-NK-92 cells, and the volume occupied by each cell population was calculated.

### Statistical analysis

Statistical analyses were performed using GraphPad Prism version 10 (GraphPad Software, San Diego, CA, USA). Data are presented as the mean ± standard error of the mean (SEM) or standard deviation (SD). Comparisons between two groups were conducted using a two-tailed unpaired t-test, whereas differences among multiple groups were evaluated using one-way analysis of variance (ANOVA) followed by Tukey's multiple comparison test. Statistical significance was defined as follows: * p < 0.05, ** p < 0.01, *** p < 0.001, and **** p < 0.0001.

## Results

### MSLN-CAR and CAR-IL-15 NK-92 cells exhibit augmented cytotoxicity and cytokine responses

**Figure 1:**
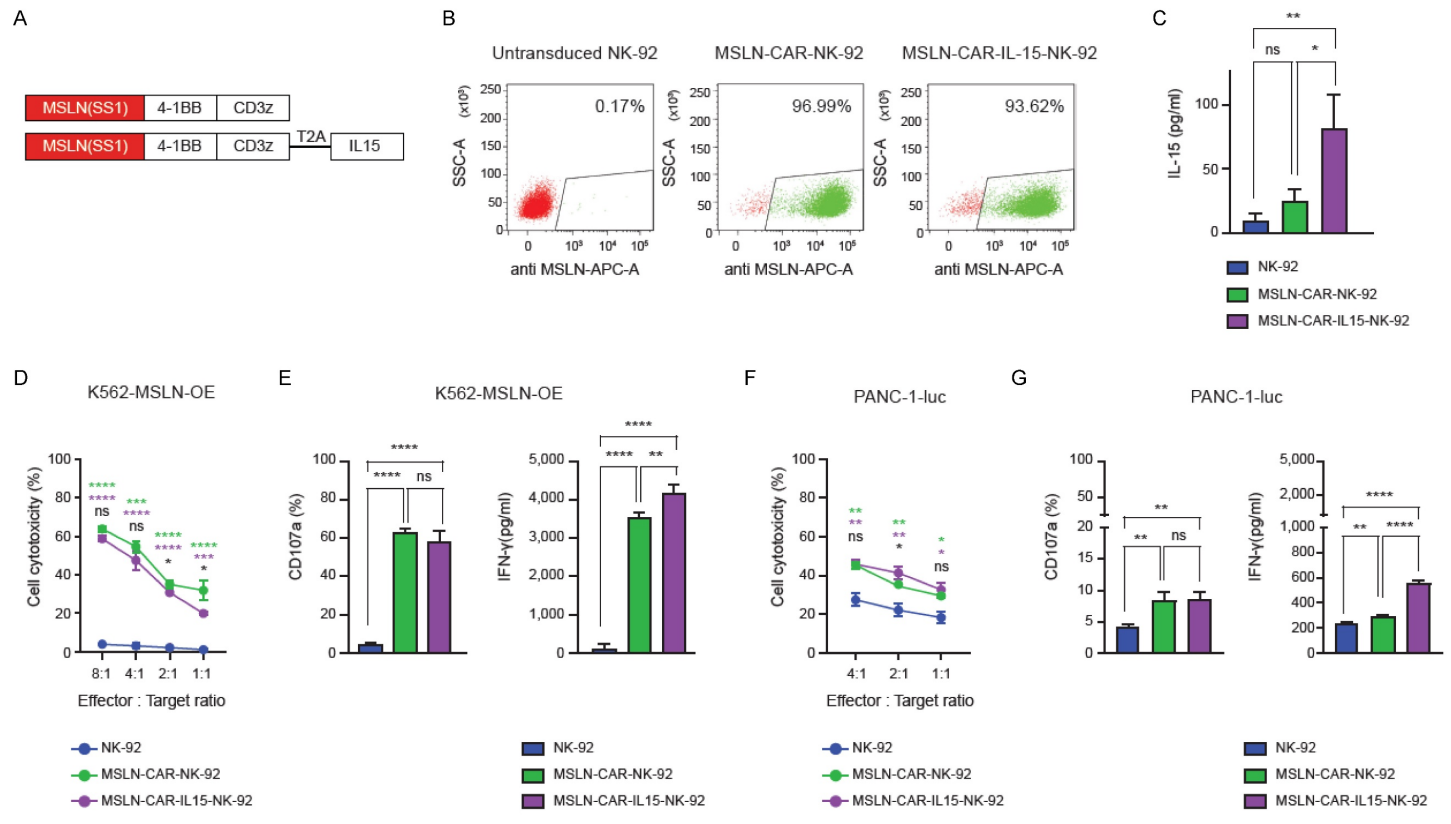
*In vitro* functional characterization of MSLN-CAR-NK-92 and MSLN-CAR-IL-15-NK-92 cells. (A) Schematic representation of the second generation MSLN-CAR constructs with or without IL-15 co-expression. (B) Flow cytometric analysis of transduction efficiency in NK-92 cells transduced with MSLN-CAR or MSLN-CAR-IL-15 constructs. (C) IL-15 secretion levels from NK-92, MSLN-CAR-NK-92, and MSLN-CAR-IL-15-NK-92 cells after 24 h of culture. (D) Cytotoxicity of NK-92, MSLN-CAR-NK-92, and MSLN-CAR-IL-15-NK-92 cells against MSLN-OE K562 cells at various effector-to-target ratios. (E) CD107a expression and IFN-γ production by NK-92, MSLN-CAR-NK-92 and MSLN-CAR-IL-15-NK-92 cells after co-culture with MSLN-OE K562 target cells. (F) Cytotoxicity of NK-92, MSLN-CAR-NK-92, and MSLN-CAR-IL-15-NK-92 cells against PANC-1-luc cells at various effector-to-target ratios. (G) CD107a expression and IFN-γ production by NK-92, MSLN-CAR-NK-92 and MSLN-CAR-IL-15-NK-92 cells after co-culture with PANC-1-luc target cells. Data are presented as mean ± standard deviation (SD). Statistical significance was assessed using unpaired t-test (D, F) and ordinary one-way ANOVA followed by Tukey's multiple comparison test (E, G) * p* <* 0.05, ** p* <* 0.01, *** p *<* 0.001, **** p *<* 0.0001. Colored asterisks indicate statistical comparisons between groups (Green: NK-92 vs. MSLN-CAR-NK-92; purple: NK-92 vs. MSLN-CAR-IL-15-NK-92; black: MSLN-CAR-NK-92 vs. MSLN-CAR-IL-15-NK-92).

MSLN-specific CAR constructs incorporating 4-1BB and CD3ζ domains were generated, with or without IL-15 co-expression via T2A linkage (Figure 1A). The transduction efficiency in NK-92 cells exceeded 90% for both constructs (Figure 1B). IL-15 secretion was detected in the MSLN-CAR-IL15-NK-92 cells (Figure 1C). In the 4 h killing assays against K562-MSLN-OE targets (Figure 1D and Figure S1A), both MSLN-CAR and MSLN-CAR-IL-15-NK-92 cells exhibited markedly higher cytotoxicity than parental NK-92 cells across all effector-to-target ratios, with no material difference between the two CAR groups. Consistently, degranulation (CD107a) and IFN-γ secretion were strongly increased in both CAR groups relative to NK-92 (Figure 1E). Against PANC-1-luc targets that endogenously expressed mesothelin (Figure 1F-G and Figure S1B), CAR engineering again enhanced killing and CD107a responses versus NK-92, although the overall cytotoxicity was lower than that against K562-mesothelin-OE. Notably, IL-15 co-expression further augmented cytokine output: MSLN-CAR-IL-15-NK-92 cells produced significantly more IFN-γ than both NK-92 and MSLN-CAR-NK-92 cells, whereas CD107a levels were comparable between the two CAR groups (Figure 1G). To assess antigen specificity, cytotoxicity and activation responses were also evaluated against mesothelin-negative target cells (K562 and Huh-7). In contrast to mesothelin-expressing targets, CAR-NK-92 cells did not exhibit enhanced cytotoxicity, degranulation (CD107a), or IFN-γ production compared with parental NK-92 cells (Figure S2).

### IL-15 co-expression enhances NK-92 cell persistence and long-term cytotoxicity

**Figure 2:**
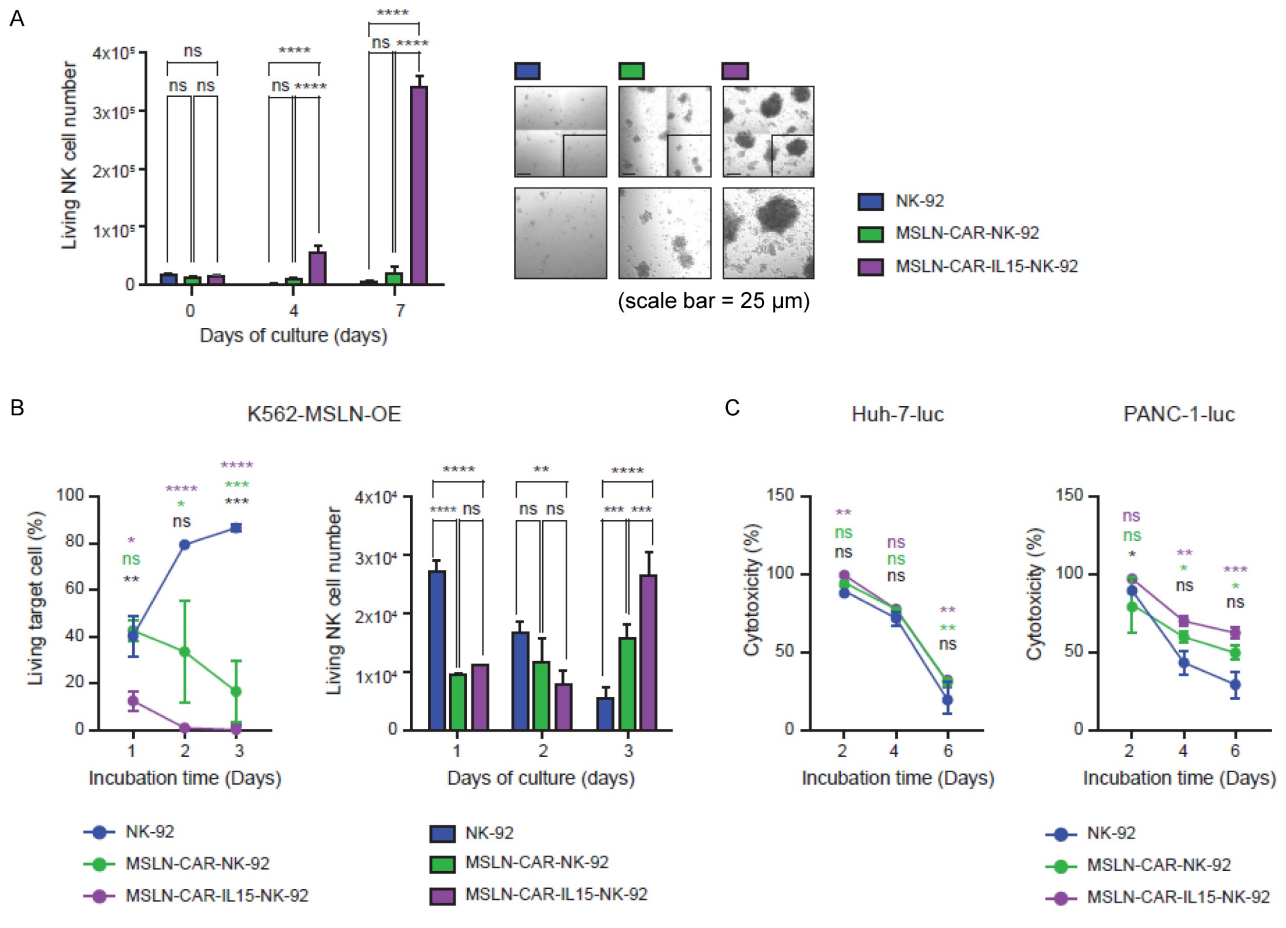
(A) Viable NK cell counts over time under cytokine-free culture conditions, with representative confocal microscopy images at Day 7. All scale bars, 25 μm. (B) Longitudinal assessment of cytotoxicity against MSLN-OE K562 target cells (left) and the number of viable NK-92 cells (right) for 3 days. (C) Serial cytotoxicity assays of NK-92, MSLN-CAR-NK-92 and MSLN-CAR-IL-15-NK-92 cells against Huh-7-luc and PANC-1-luc target cells at an E:T ratio of 4:1. Data are presented as mean ± standard deviation (SD). Statistical significance was assessed using unpaired t-test (B-left) and ordinary one-way ANOVA followed by Tukey's multiple comparison test (B-right, C) * p *<* 0.05, ** p *<* 0.01, *** p *<* 0.001, **** p *<* 0.0001. Colored asterisks indicate statistical comparisons between groups (Green: NK-92 vs. MSLN-CAR-NK-92; purple: NK-92 vs. MSLN-CAR-IL-15-NK-92; black: MSLN-CAR-NK-92 vs. MSLN-CAR-IL-15-NK-92).

Before evaluating persistence and long-term cytotoxicity, we examined whether CAR expression or IL-15 co-expression altered the activation phenotype of NK-92 cells. The expression of NKG2D, NKp44, and CD69 were analyzed at 24, 48, and 72 h by flow cytometry, and no notable differences were observed among NK-92, MSLN-CAR-NK-92, and MSLN-CAR-IL-15-NK-92 cells (Figure S3). To assess whether IL-15 co-expression supported NK-92 persistence in the absence of exogenous cytokines, NK-92, MSLN-CAR-NK-92, and MSLN-CAR-IL-15-NK-92 cells were cultured without IL-2. MSLN-CAR-IL-15-NK-92 cells showed the most pronounced expansion by day 7, exceeding that of both parental and MSLN-CAR-NK-92 cells (Figure 2A). Under the same cytokine-free conditions, serial co-culture with K562-mesothelin-OE targets demonstrated that parental NK-92 and MSLN-CAR-NK-92 cells progressively lost activity, whereas MSLN-CAR-IL-15-NK-92 cells maintained a low percentage of living targets and increased their own cell numbers by day three, indicating IL-15-driven persistence during repeated engagements (Figure 2B). In the 6-day serial killing assays against tumor lines, cytotoxicity was comparable across groups of mesothelin-negative Huh-7-luc cells (Figure 2C, left). In contrast, in mesothelin-positive PANC-1-luc cells, the parental and MSLN-CAR-NK-92 cells rapidly declined in activity, whereas MSLN-CAR-IL-15-NK-92 cells sustained robust killing over time (Figure 2C, right), underscoring the role of IL-15 in preserving the serial killing capacity of antigen-positive targets.

### Confirmation of mesothelin expression in human pancreatic cancer cell line, PANC-1, xenografts

**Figure 3:**
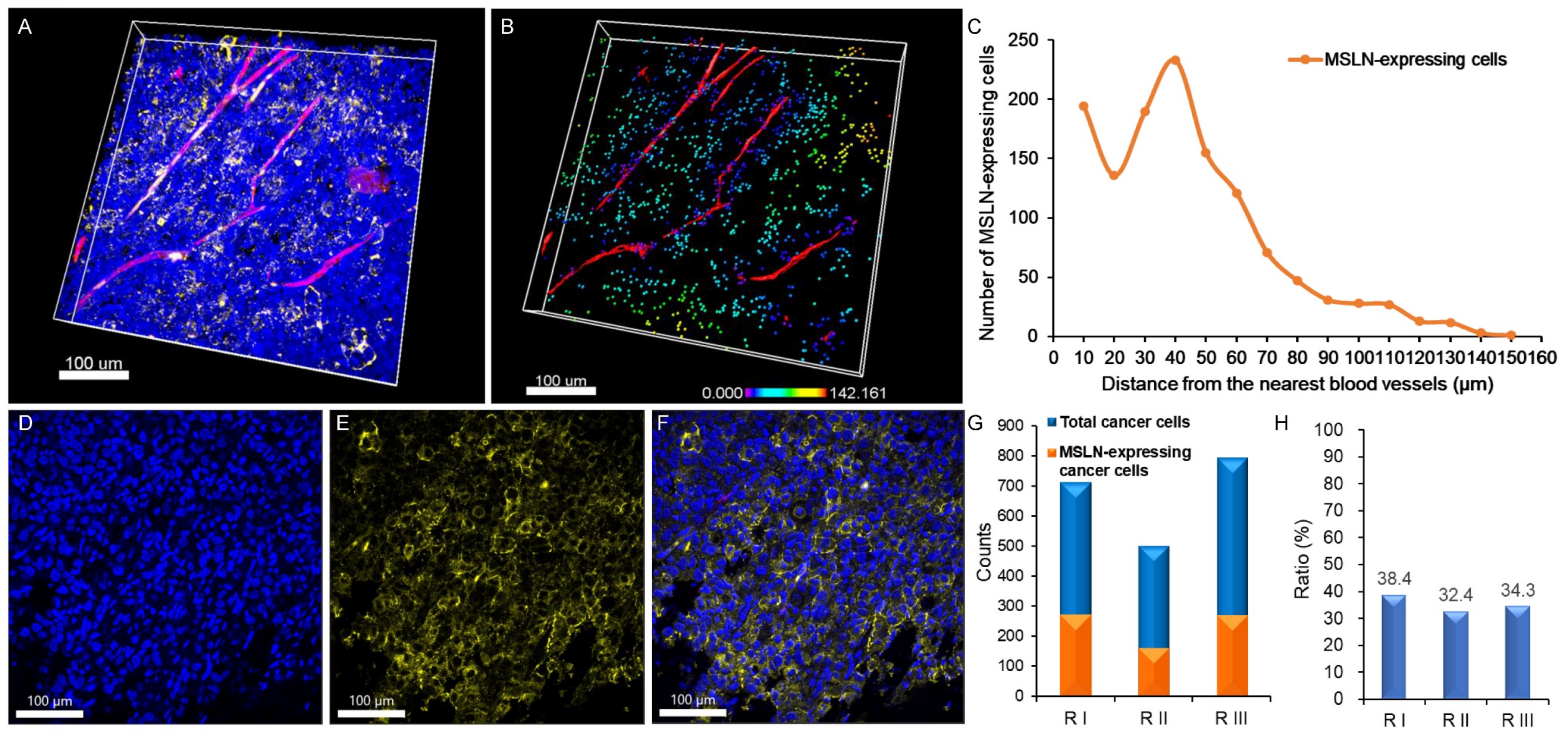
Expression of mesothelin (MSLN) in PANC-1 pancreatic cancer xenografts. (A) 3D image showing nuclei (blue), blood vessels (red), and MSLN expression (yellow) in PANC-1 pancreatic tumors. (B) 3D-reconstructed image based on (A) (raw image). MSLN-expressing cancer cells are represented by spectral colors based on their distance from the nearest blood vessel. (C) Distribution of MSLN-expressing cancer cells relative to the nearest blood vessel. (D-F) 2D section images showing (D) nuclei, (E) MSLN expression, and (F) merged image of nuclei and MSLN. (G) Number of MSLN-expressing cancer cells compared to the total cancer cells in the three different regions. (H) Proportion (%) of MSLN-expressing cancer cells relative to total cancer cells in the three different regions. (n = 3 independent biological experiments). All scale bars, 100 μm.

Mesothelin expression was evaluated in PANC-1 xenografts derived from NSG mice. The tumor tissue was processed by optical clearing and subjected to 3D imaging. The raw transparent tumor image (Figure 3A) and its 3D-reconstructed counterpart (Figure 3B) demonstrate the presence and spatial distribution of mesothelin-positive cancer cells. A representative single-plane image further confirmed the mesothelin localization on the outer membrane of the tumor cells (Figure 3D-F). Quantitative analysis across three independent tumor regions showed that 273/711, 162/500, and 271/791 cells expressed mesothelin, corresponding to 38.4%, 32.4%, and 34.4% positivity, respectively (Figure 3G-H). These results indicate that, whereas only ~5-6% of PANC-1 cells expressed mesothelin under in vitro 2D culture conditions, a substantially higher proportion (30 - 40%) of tumor cells expressed mesothelin within the xenograft model.

### Spatial distribution of NK-92-GFP and MSLN-CAR-NK-92-GFP cells in PANC-1 xenografts

**Figure 4:**
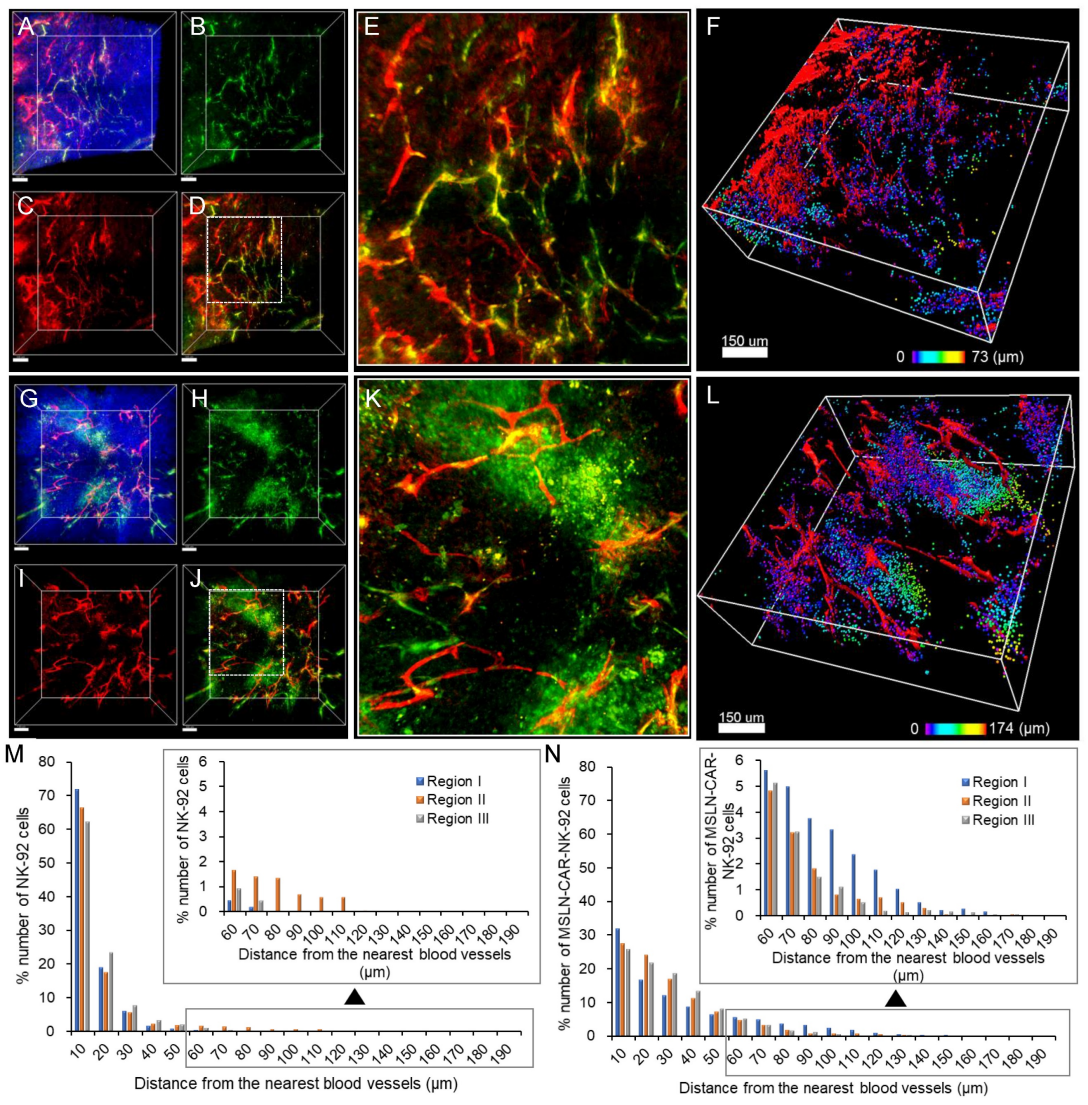
3D tumor images showing the spatial distribution of (A-F) NK-92-GFP cells and (G-L) MSLN-CAR-NK-92-GFP cells in pancreatic cancer. (A) Merged image of blood vessels and apoptotic cells with nuclei labeled with Hoechst. (B-C) 3D images of (B) NK-92-GFP cells and (C) blood vessels. (D) Merged image of the NK-92-GFP cells and blood vessels. (E) Magnified image of white boxed area in image (D). (F) 3D-reconstructed image of (A) (raw image). Spots (NK-92-GFP cells) are represented in spectral color based on their distance to the nearest blood vessels. (G) Merged image of blood vessels and MSLN-CAR-NK-92-GFP cells with nuclei labeled with Hoechst. (H-I) 3D images of (H) MSLN-CAR-NK-92-GFP cells and (I) blood vessels. (J) Merged image of the MSLN-CAR-NK-92-GFP cells and blood vessels. (K) Magnified image of white boxed area in image (K). (L) 3D-reconstructed image of (G) (raw image). Spots (MSLN-CAR-NK-92-GFP cells) are represented in spectral color based on their distance to the nearest blood vessel. (M-N) Graphical data signify the percentage number of (M) NK-92 cells and (N) MSLN-CAR-NK-92-GFP cells that were distributed in relation to the distance from the blood vessel. (n = 3 independent biological experiments). Scale bars, (A-D) and (G-J) 100 μm, (F) and (L) 150 μm.

Given the confirmed mesothelin expression in PANC-1 xenografts (Figure 3), we compared the intratumoral distribution of NK-92-GFP and MSLN-CAR-NK-92-GFP cells after their intravenous administration into NSG mice. Transparent tumor imaging revealed that NK-92-GFP cells were largely confined to blood vessels and their immediate surroundings (Figure 4A-E). In the reconstituted 3D image (Figure 4F), NK-92-GFP cells were detected mainly within vascular regions, indicating limited penetration of the tumor tissue. In contrast, MSLN-CAR-NK-92-GFP cells displayed a broader dispersion pattern (Figure 4G-K). Although some cells remained within the blood vessels, many were observed beyond the vasculature, as highlighted in the reconstituted 3D images (Figure 4I). Quantitative distance mapping demonstrated that NK-92-GFP cells extended up to ~73 μm from blood vessels, whereas MSLN-CAR-NK-92-GFP cells migrated as far as ~174 μm (Figure 4M-N). Analysis across three independent regions confirmed that both groups showed a gradual decrease in cell numbers with increasing distance, but CAR-engineered cells consistently exhibited greater migration and distribution, despite equal input numbers. Consistent with the full cross-sectional confocal mosaic images (Figure S4), MSLN-CAR-NK-92-GFP cells exhibited a broader and more extensive intratumoral distribution compared to NK-92-GFP cells. This observation suggests enhanced tumor infiltration and extravasation capacity of MSLN-CAR-engineered NK cells within the heterogeneous tumor microenvironment. These results indicate that MSLN-CAR modification enhances the ability of NK-92 cells to extravasate and infiltrate tumor tissue *in vivo*, complementing the higher mesothelin levels observed in PANC-1 xenografts compared to *in vitro* cultures.

### Spatial distribution of NK-92 and MSLN-CAR-NK-92 cell-induced apoptotic cell death in PANC-1 xenografts

**Figure 5:**
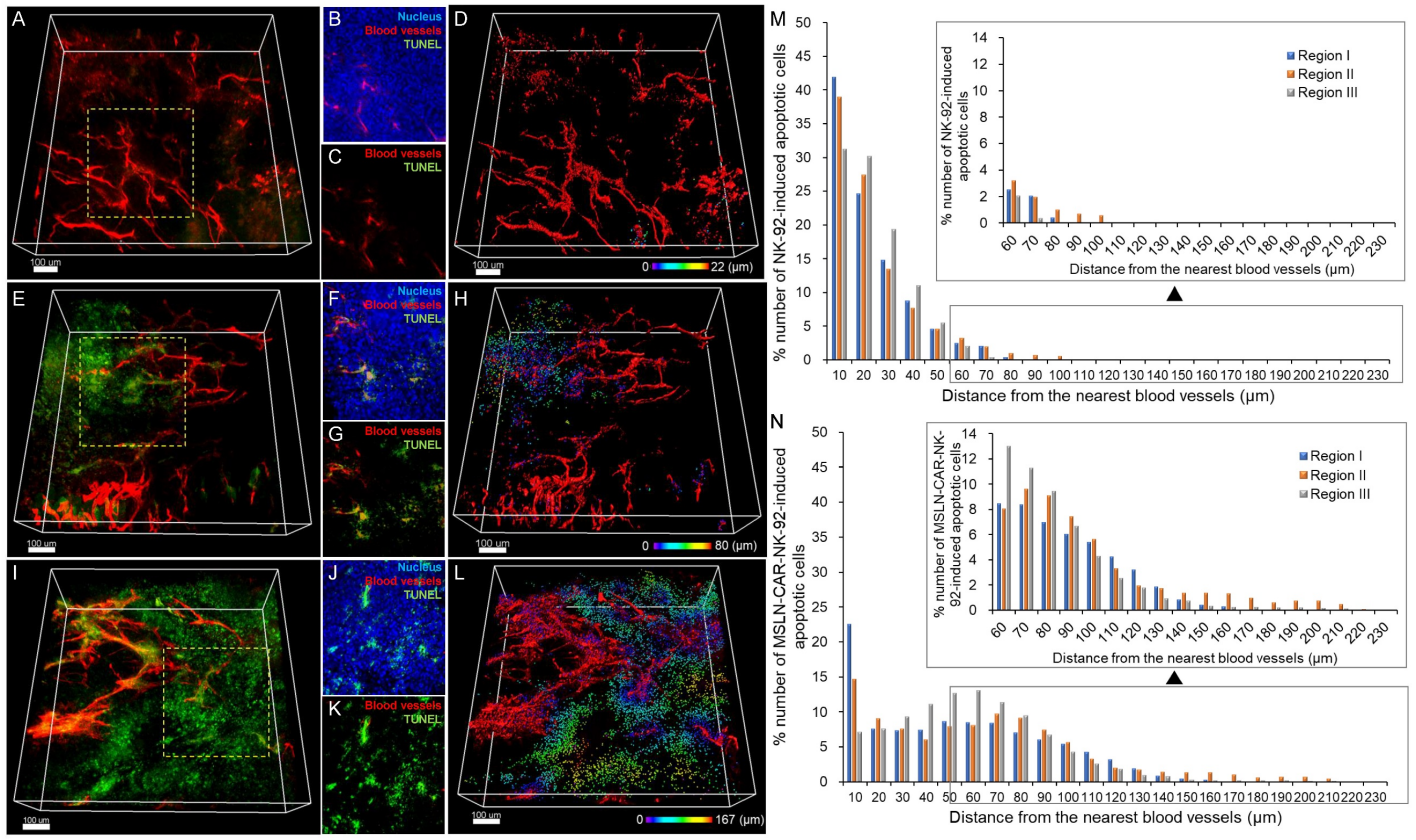
3D tumor imaging showing the spatial distribution of NK-92 and MSLN-CAR-IL-15-NK-92-induced apoptotic cell death in pancreatic cancer. (A-D) Control group. (E-H) NK-92-treated group. (I-L) MSLN-CAR-IL-15-NK-92-treated group. (M) Graph showing the percentage of NK-92-induced apoptotic pancreatic cancer cells based on their distance to the nearest blood vessel. (N) Graph showing the percentage of MSLN-CAR-IL-15-NK-92-induced apoptotic pancreatic cancer cells based on their distance to the nearest blood vessel. (n = 3 independent biological experiments). All scale bars, 100 μm.

To further determine whether the enhanced intratumoral distribution of CAR-engineered NK cells translated into increased cytotoxic activity, we evaluated the *in vivo* apoptosis of tumor cells after treatment with NK-92 or MSLN-CAR-IL15-NK-92 cells. Transparent tumor imaging revealed minimal cell death in the control group (Figure 5A-C). In contrast, NK-92 cell treatment induced notable tumor cell apoptosis, particularly in regions adjacent to the blood vessels (Figure 5E-G). Importantly, MSLN-CAR-IL-15-NK-92 cell treatment resulted in substantially greater apoptosis, with apoptotic signals extending farther from the blood vessels compared to the NK-92 treatment (Figure 5I-K). 3D reconstruction-based distance mapping confirmed that apoptotic cell distribution extended up to 22 μm in the control, 80 μm in NK-92-treated tumors, and 167 μm in MSLN-CAR-IL-15-NK-92-treated tumors (Figure 5D, 5H and 5I). Quantitative analysis of multiple tumor regions further demonstrated that MSLN-CAR-IL-15-NK-92 cells induced broader and more sustained tumor cell death at a greater distance from the blood vessels than NK-92 cells (Figure 5M-N). To further validate and quantify the extent of intratumoral penetration and apoptotic cell death induced by MSLN-CAR-NK-92 cells, we analyzed the cell distribution and apoptosis depth using cumulative distance mapping.

### Comparison of distribution and efficacy of NK-92 and MSLN-CAR-NK-92 cells in human pancreatic cancer xenografts

**Figure 6:**
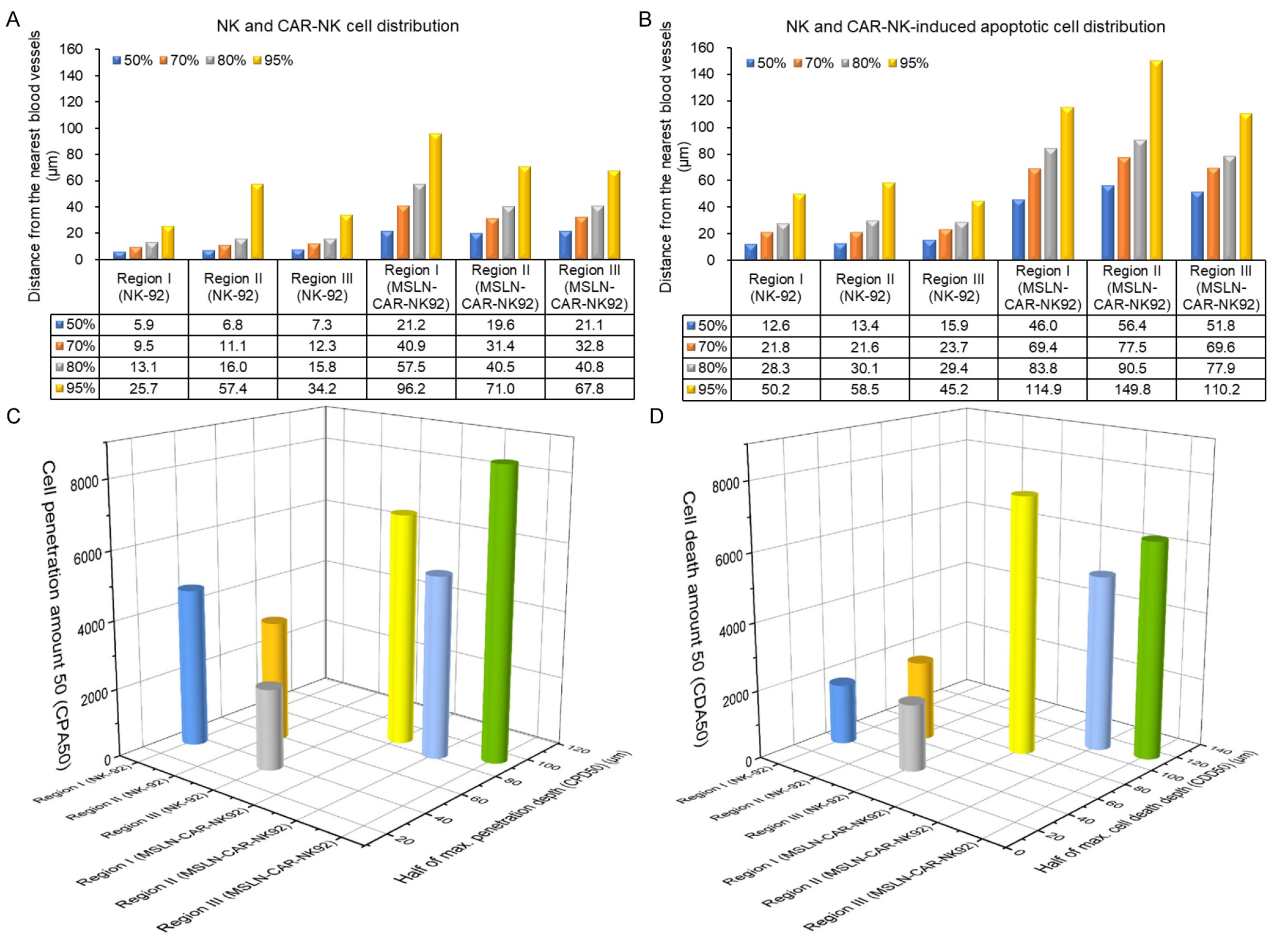
(A and B) Graphs presenting the cumulative percentage of (A) distribution of NK-92 and MSLN-CAR-NK-92 cells and (B) NK-92 and MSLN-CAR-NK-92 cell-induced apoptotic cancer cells, respectively, as a function of distance from the nearest blood vessel. (C) 3D plots of NK-92 and MSLN-CAR-NK-92 cell penetration amount 50 (CPA50) and half of the maximum cell penetration depth (CPD50). (D) 3D plots of cell death amount 50 (CDA50) and half of the maximum cell death depth (CDD50) induced by NK-92 and MSLN-CAR-NK-92 cells. (n = 3 independent biological experiments).

The escape distance corresponding to the cumulative 95% of NK-92 cells was approximately 26 - 57 µm, whereas that of MSLN-CAR-NK-92 cells ranged from 68 to 96 µm (Figure 6A). The escape distance for MSLN-CAR-NK-92 cells increased by approximately 39 - 70 µm compared to that of NK-92 cells (Figure 6A). The distribution of apoptotic cell death induced by NK-92 and MSLN-CAR-NK-92 cells shows that the cumulative 95% of NK-92-induced apoptotic cell death is distributed within 45 - 59 µm, while the cumulative 95% of MSLN-CAR-NK-92-induced apoptotic cell death extends to 110 - 150 µm (Figure 6B). The distribution of apoptotic cell death caused by MSLN-CAR-NK-92 cells extends 51 - 105 µm farther than that induced by NK-92 cells (Figure 6B). In this study, half of the maximum cell penetration depth (CPD50) and the number of NK-92 or MSLN-CAR-NK-92 cells from the intratumoral blood vessels to the CPD50 (referred to as the cell penetration amount 50 or CPA50) were measured (Figure 6C). For NK-92 cells, the CPA50 values corresponding to CPD50 in the three different regions were 37 µm - 4627 cells, 60 µm - 3537 cells, and 37 µm - 2364 cells. In the case of MSLN-CAR-NK-92 cells, the CPA50 values corresponding to CPD50 in the three different regions were 87 µm - 6790 cells, 83 µm - 5344 cells, and 93 µm - 8528 cells. On average, MSLN-CAR-NK-92 cells (88 µm - 6887 cells) showed approximately a twofold improvement in both CPA50 and CPD50 compared to NK-92 cells (45 µm - 3509 cells). In addition, we analyzed half of the maximum cell death depth (CDD50) and the number of apoptotic cell deaths from the intratumoral blood vessels to CDD50 (referred to as the cell death amount 50 or CDA50) (Figure 6D). For NK-92 cells, the CDA50 values corresponding to CDD50 in the three different regions were 40 µm - 1773 cells, 67 µm - 2338 cells, and 36 µm - 1958 cells. In the case of MSLN-CAR-NK-92 cells, the CDA50 values corresponding to CDD50 in the three different regions were 83 µm - 7527 cells, 109 µm - 5164 cells, and 114 µm - 6360 cells. On average, cell death induced by MSLN-CAR-NK-92 cells (102 µm - 6350 cells) showed approximately a threefold increase in CDA50 and a twofold increase in CDD50 compared to cell death induced by NK-92 cells (48 µm - 2023 cells).

### Comparison of spatial distribution of NK-92 and MSLN-CAR-NK-92 cells following co-injection in human pancreatic cancer xenografts

**Figure 7:**
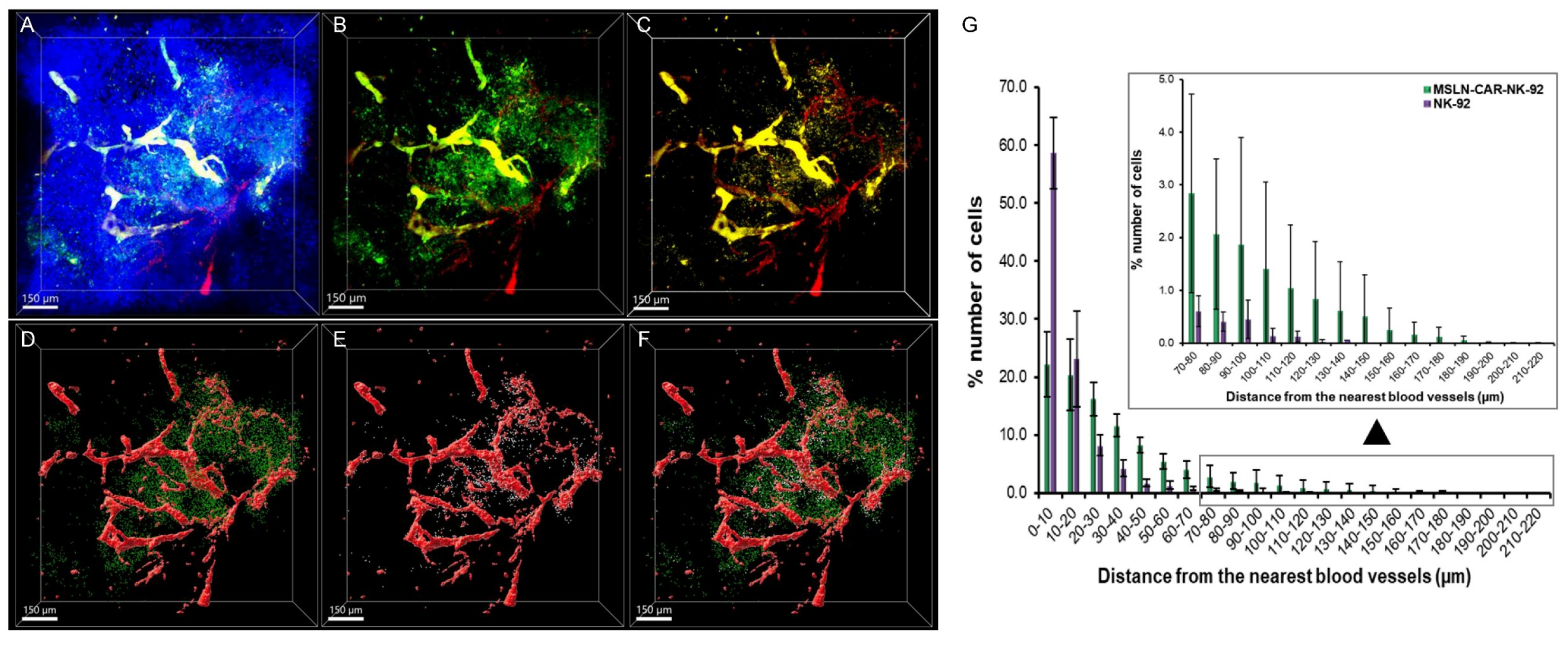
Spatial distribution of NK-92 (CellTrace™-labeled) and MSLN-CAR-NK-92-GFP cells after simultaneous administration in PANC-1 pancreatic cancer xenografts. 3D images showing nuclei (blue), blood vessels (red), MSLN-CAR-NK-92 cells (green) and NK-92 cells (yellow) in PANC-1 pancreatic tumors. (A) Merged 3D image of blood vessels, MSLN-CAR-NK-92 cells and NK-92 cells with nuclei labeled with Hoechst. (B-C) 3D image of (B) MSLN-CAR-NK-92 cells with blood vessels and (C) NK-92 cells with blood vessels. (D) 3D-reconstructed image of (B) (raw image) represents MSLN-CAR-NK-92 cell spots (green) and vasculature (red). (E) 3D-reconstructed image of (C) (raw image) represents NK-92 cell spots (yellow) and vasculature (red). (F) MSLN-CAR-NK-92 cells, NK-92 cells and blood vessels are represented together as green spots, yellow spots, and red structures, respectively. (G) Graph showing the percentage of NK-92 cells and MSLN-CAR-NK-92 cells as a function of distance to the nearest blood vessel. (n = 3 independent biological experiments). Data are presented as mean ± standard deviation (SD). All scale bars, 150 μm.

As shown in Figure 4 and 5, we demonstrated that MSLN-CAR-NK-92 cells not only infiltrated tumor tissue more effectively than parental NK-92 cells, but also induced more extensive apoptosis when administered individually into separate mice. To directly validate these differences under identical *in vivo* conditions, we performed co-injection experiments in which NK-92 and MSLN-CAR-NK-92 cells were simultaneously intravenously administered at equal cell numbers into the same pancreatic cancer xenograft-bearing mice. MSLN-CAR-NK-92 cells expressing GFP (488 nm excitation) and NK-92 cells stained with CellTrace™ (561 nm excitation) were used. The signals were separated by distinct excitation/emission settings, allowing simultaneous intratumoral 3D visualization of both populations. As shown in Figure 7A, z-stack imaging through ~300 μm thickness revealed tumor neclei (blue), vasculature (red), NK-92 cells (yellow), and MSLN-CAR-NK-92 cells (green). Separate channel visualization further highlighted the distribution of MSLN-CAR-NK-92 cells relative to vasculature (Figure 7B) and NK-92 cells relative to the vasculature (Figure 7C). Despite equal co-injection, MSLN-CAR-NK-92 cells displayed a markedly deeper and broader intratumoral distribution than NK-92 cells. The 3D reconstruction of the three distinct tumor regions further confirmed this difference (Figure 7D-F). In spot-based spatial analysis, NK-92 cells (white spots) were mainly restricted to the perivascular regions, whereas MSLN-CAR-NK-92 cells (green spots) penetrated farther away from the blood vessels (red). Quantitative analysis across three tumor regions (Figure 7G) showed that while both populations exhibited a decay with increasing distance from vasculature, MSLN-CAR-NK-92 cells achieved significantly greater maximum penetration (185 ± 22 μm) compared to NK-92 cells (128 ± 7 μm). The total number of extravasated MSLN-CAR-NK-92 cells was approximately 3.3 fold greater than that of NK-92 cells (7,717 vs. 2,311 cells). Collectively, these results show that MSLN-CAR-NK-92 cells achieve deeper intratumoral penetration and extravasate more efficiently from the tumor-associated vasculature. This enhanced trafficking likely contributes to their superior capacity to infiltrate and exert cytotoxic effects within tumor tissues, reinforcing the antitumor potential of CAR engineering.

### Quantitative evaluation of extravasated NK-92 and MSLN-CAR-NK-92 cells

**Figure 8:**
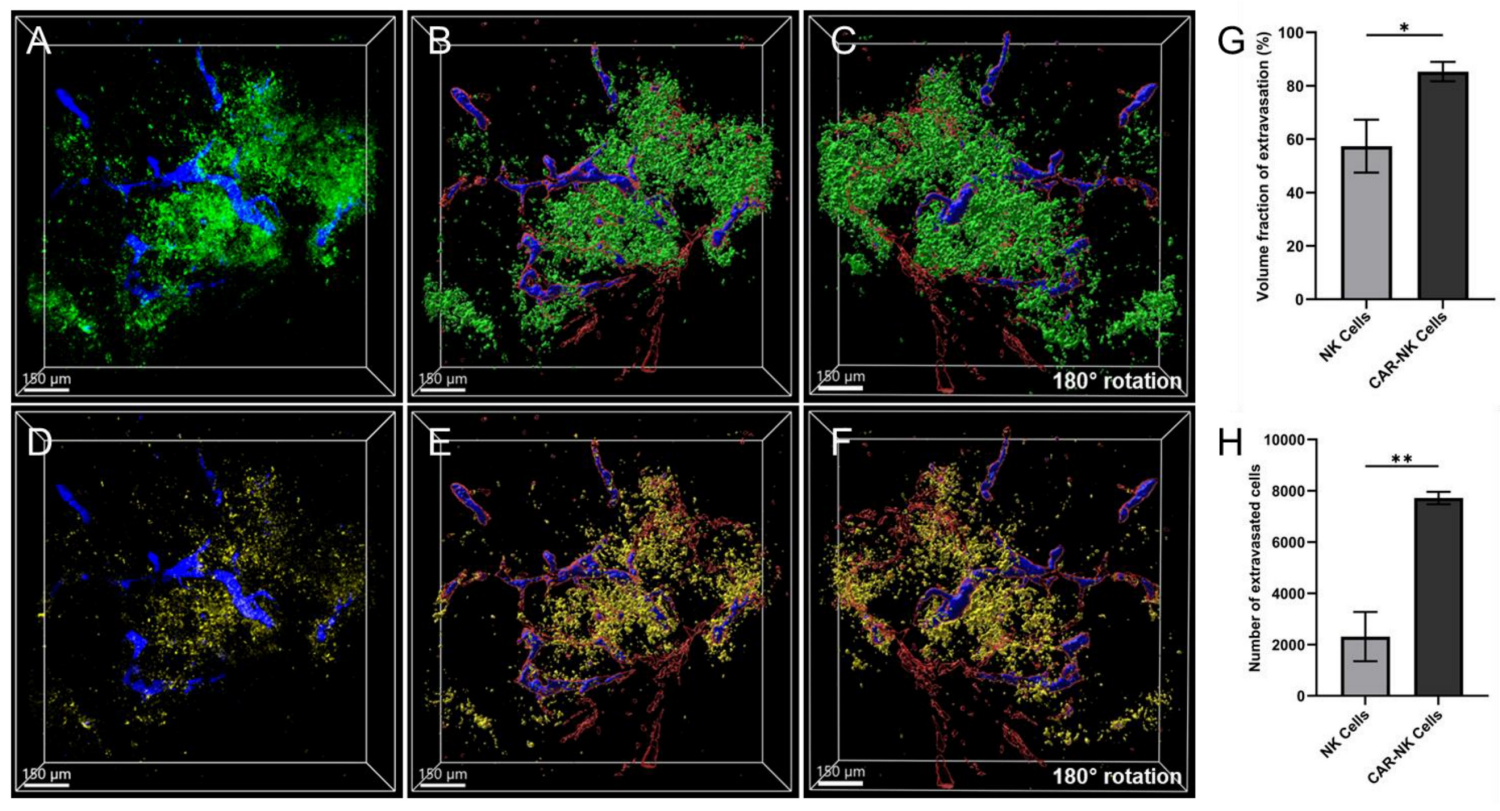
Quantitative analysis of extravasation degree of NK-92 and MSLN-CAR-NK-92-GFP cells after simultaneous administration in PANC-1 pancreatic cancer xenografts. (A) Merged 3D image of extravasated (green) and intravascular (blue) MSLN-CAR-NK-92 cells. (B) 3D-reconstructed image represents extravasated (green) and intravascular (blue) MSLN-CAR-NK-92 cells and vasculature (red). (C) represents a 180° rotation of image (B). (D) Merged 3D image of extravasated (yellow) and intravascular (blue) NK-92 cells. (E) 3D-reconstructed image represents extravasated (yellow) and intravascular (blue) NK-92 cells and vasculature (red). (F) represents a 180° rotation of image (E). (G) Graph illustrating the extravasation volume fraction of NK-92 and MSLN-CAR-NK-92 cells. (H) Graph showing the number of extravasated cells of NK-92 cells and MSLN-CAR-NK-92 cells. Data are presented as mean ± SD (n = 3); * p *<* 0.05, ** p *<* 0.01. (n = 3 independent biological experiments). All scale bars, 150 μm.

Next, we quantitatively compared the extent of extravasation between NK-92 and MSLN-CAR-NK-92 cells to evaluate their ability to reach and target the tumor tissue. Intravenously administered CAR NK cells circulate through the vasculature and initiate rolling interactions within the TME by binding to endothelial adhesion molecules that are overexpressed in tumor-associated blood vessels. This rolling is mediated by the interaction between adhesion molecules (e.g., ICAM-1 and VCAM-1) and integrins on the NK cell surface. Upon activation, these integrins undergo conformational changes into a high-affinity state, leading to firm adhesion and subsequent transendothelial migration via the paracellular route 19-22. This well-characterized mechanism of immune cell extravasation has been extensively studied and is known to facilitate immune cell infiltration of solid tumors 23. Fluorescent signals that co-localized with the vasculature were classified as immune cells existing in the blood vessels. In contrast, immune cells positioned outside the vascular structures without overlapping fluorescence were classified as extravascular cells that had extravasated into the tumor tissue. In the image showing the distribution of MSLN-CAR-NK-92 cells and the vasculature (Figure 7B), CAR-NK cells located within the vasculature were identified. The corresponding segmented and 3D-reconstructed image derived from the dataset shown in Figure 7B is presented in Figure 8A, in which intravascular CAR-NK cells are visualized in blue, whereas cells that had extravasated beyond the vessel boundaries are displayed in green. Similarly, in the image showing NK-92 cells and vasculature (Figure 7C), intravascular NK-92 cells were identified. The reconstructed and segmented image generated from the dataset in Figure 7C is shown in Figure 8D, where intravascular NK-92 cells are visualized in blue and extravascular NK-92 cells are represented in yellow. A comparison of Figure 8A-D demonstrates that although MSLN-CAR-NK-92 and NK-92 cells were not uniformly present within all tumor-associated blood vessels, they were densely packed within specific vascular regions. From these areas of accumulation, the cells were observed to extravasate into the perivascular tumor tissue. Notably, MSLN-CAR-NK-92 cells were found to localize within a greater number of vascular regions than NK-92 cells and exhibited a higher degree of extravasation, migrating farther into the tumor tissue. To quantitatively assess the extent of NK cell extravasation, the intravascular and extravascular cells were reconstructed independently in 3D. As shown in Figure 8B, blue-colored intravascular MSLN-CAR-NK-92 cells were localized within the blood vessels outlined in red, whereas green-colored extravascular MSLN-CAR-NK-92 cells were widely dispersed throughout the surrounding tumor tissue. This distribution was also evident in the 180-degree rotated view from the bottom, further illustrating the three-dimensional localization of MSLN-CAR-NK-92 cells around the vasculature (Figure 8C). Similarly, the 3D reconstruction of NK-92 cells showed blue-colored intravascular NK-92 cells located within blood vessels, outlined in red, along with yellow-colored extravascular NK-92 cells distributed outside the vasculature (Figure 8E). The 180-degree rotated view further demonstrates the three-dimensional perivascular distribution of NK-92 cells, highlighting their intrinsic anti-tumor capability as innate immune cells (Figure 8F). The relative degree of extravasation was quantitatively analyzed by measuring the volume occupied by intravascular and extravascular NK cells in the 3D reconstructed images (Figure 8B and 8E). The volume fraction of the extravasated NK cells was calculated using the following formula:

Relative extravasation fraction (%) = 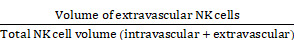
× 100

NK-92 cells exhibited an extravasation fraction of 57.4%, whereas MSLN-CAR-NK-92 cells exhibited a significantly higher extravasation fraction (85.3%). Despite the substantial number of cells remaining intravascular, the CAR-engineered cells demonstrated markedly enhanced extravasation, resulting in a greater proportion of cells migrating out of the vasculature (Figure 8G). Furthermore, the number of extravasated cells was 7,717 for MSLN-CAR-NK-92 cells, which was 3.3 times higher than that 2,311 observed in NK-92 cells (Figure 8H). Taken together, MSLN-CAR-NK-92 cells were more efficiently recruited to the tumor-associated vasculature than parental NK-92 cells and exhibited greater extravasation in both quantity and distance. These findings highlight the enhanced antitumor potential of CAR engineering.

Next, to determine whether IL-15 secretion influences the migration and infiltration capacity of CAR-NK-92 cells *in vivo*, the distribution of MSLN-CAR-IL-15-NK-92-GFP cells was compared with that of MSLN-CAR-NK-92-GFP cells (Figure S5). The volume fraction of extravasation was 86.3 ± 1.5% for MSLN-CAR-IL-15-NK-92-GFP cells and 85.3 ± 3.0% for MSLN-CAR-NK-92-GFP cells. The number of extravasated cells was 7,886 ± 306 and 7,717 ± 200 cells, respectively, showing no significant difference in cell distribution patterns between the two groups. These results indicate that IL-15 secretion does not substantially affect the migration or infiltration behavior of CAR-NK-92 cells *in vivo*, suggesting that the enhanced extravasation observed in this study is primarily associated with CAR-mediated tumor targeting.

To further examine whether the proposed imaging and quantitative analysis approach can be applied to another solid tumor model, we performed additional experiments using a HuCCT1 cholangiocarcinoma xenograft model (Figure S6). The intratumoral distribution of MSLN-CAR-NK-92-GFP cells was analyzed using the same 3D imaging and analysis methods. Quantitative analysis showed that the maximum penetration distance from the nearest blood vessel was 145.4 ± 0.9 μm in HuCCT1 tumors, which was lower than that observed in PANC-1 tumors (184.9 ± 22.3 μm). However, the number of extravasated cells was comparable between the two tumor models (7717 ± 200 cells in PANC-1 vs. 7474 ± 1145 cells in HuCCT1 tumors). Similarly, the volume fraction of extravasated cells was 85.3 ± 3.0% in PANC-1 tumors and 81.0 ± 4.7% in HuCCT1 tumors. These results indicate that the quantitative analysis results vary depending on tumor type, the extravasation efficiency and intratumoral distribution of MSLN-CAR-NK-92 cells can be consistently quantified using the same imaging and analysis workflow. This supports the applicability of the proposed quantitative methodology for evaluating immune cell delivery and spatial distribution within the tumor microenvironment.

## Discussion

To the best of our knowledge, this study provides the first microscale, 3D, vessel-preserving quantification of immune cell extravasation and parenchymal penetration in a solid tumor setting using tissue clearing of pancreatic cancer xenografts. Mesothelin (MSLN) targeting by second-generation CARs augmented acute effector function *in vitro* and, critically translated *in vivo* into greater vascular egress, deeper intratumoral dispersion, and broader apoptotic footprints compared with parental NK-92 cells. Quantitative indices—extravasation fraction, CPA50/CPD50 (cell penetration amount/depth at 50%), and CDA50/CDD50 (cell death amount/depth at 50%)—linked trafficking to function and enabled head-to-head construct comparison. *In vitro*, MSLN-targeted CAR NK-92 cells (SS1 scFv) outperformed parental NK-92 on mesothelin⁺ targets, with higher cytotoxicity and cytokine release 24. On mesothelin-OE K562, both MSLN-CAR and MSLN-CAR-IL-15-NK-92 cells exceeded NK-92 cells across E:T ratios with concordant CD107a and IFN-γ increases. On endogenous mesothelin⁺ PANC-1-luc, overall killing was lower—consistent with modest antigen density and stromal constraints—yet still superior to NK-92 cells. IL-15 coexpression enhances persistence under cytokine deprivation: without exogenous IL-2, CAR-IL-15-NK-92 cells expanded the most by day 7 and maintained control through repeated challenges, whereas NK-92 and non-IL-15 CAR cells waned. Over six days, groups were similar on mesothelin⁻ Huh-7-luc, but only the IL-15-armed product sustained killing on mesothelin⁺ PANC-1-luc. IL-15 chiefly increased IFN-γ without altering CD107a, implying improved metabolic/secretory fitness that preserves serial killing. These patterns mirror those of previous SS1-based MSLN-CAR reports 25-28. Given its clinical relevance, we used PANC-1-luc for *in vivo* experiments despite its lower *in vitro* susceptibility than K562-mesothelin-OE. Extravasation—the process by which immune cells in blood vessels cross the vascular endothelium to reach distant organs. Understanding how extravasation occurs is important for understanding the human immune system, and many studies have been conducted on this issue 29-31. As mainly understood until now, extravasation is a multistep process. First, immune cells adhere to the vascular wall and roll along the endothelium by upregulating adhesion receptors on vascular endothelial cells. Capture and rolling by selectins, strong binding by ICAM-1 and VCAM-1, occur in immune cells during this process. Rolling reduces the speed of immune cells. When immune cells reach the extravasation site, they use integrins to adhere more strongly to the endothelium. For immune cells to escape into the target organ, a morphological change occurs in the vascular endothelium, allowing them to cross vascular endothelial cells via the paracellular route. A new finding of the present tumor transparency imaging study was that the number of NK-92 and MSLN-CAR-NK-92 cells extravasating the pancreatic tumor was limited. Tumor transparency imaging was performed using a cell tracker and GFP expression to precisely measure the three-dimensional distribution of NK-92 (cell tracker) and MSLN-CAR-NK-92-GFP cells in the tumor on a micrometer scale as a function of the distance from the tumor blood vessels. Thus, it was possible to accurately measure the number and distribution of extravasated immune cells. Notably, the number of MSLN-CAR-NK-92 cells that extravasated to the entire tumor was much lower than expected, considering that they should be used for anticancer cell therapy. In a previous study, tumor transparency imaging was used to successfully determine the intratumor drug permeability and drug permeation amount of anticancer drugs, such as small molecules and nano drug delivery systems, by counting the number of cancer cells that take up the anticancer drug at the single-cell level 10-12. The results of this study showed that, like inanimate small molecules and nano drug delivery systems, CAR-NK cells did not spread throughout the tumor and mostly remained around the tumor blood vessels. This was an unexpected result considering the characteristics of anticancer cell therapeutic agents that exert anticancer immunity on their own as living organisms. We cannot help but consider the specific TME of solid tumors as the cause of the limited extravasation of MSLN-CAR-NK-92 and NK-92 cells. When considering the TME of solid tumors, one of the most characteristic phenomena is the overexpression of ECM. ECM is an essential element for organizing the organs that exist among cells. It is mainly produced by fibroblasts and is largely composed of fibrous proteins such as collagen, elastin, and hyaluronic acid. This ECM protein was particularly overexpressed in the pancreatic TME. Therefore, the drug permeabilities of existing compounds and nano-drug delivery systems are extremely limited 32-36. In the TME, the phenotype of the fibroblasts changes to cancer-associated fibroblasts (CAFs) through polarization. The ECM produced by these CAFs provides anticancer drugs with spatial specificity that inhibits their spread throughout the tumor. First, anticancer small molecules tend to adhere well to ECM collagen. The ECM has a tortuous shape, which prevents anticancer drugs from freely diffusing. Additionally, the overexpressed ECM is densely distributed around cancer cells and tends to isolate cancer cells. Consequently, intratumoral spaces are created, making it more difficult for anticancer drugs to access the cancer cells 34, 37-39. In this study, we hypothesized that cancer-friendly ECM expression exerts a strong influence on NK-92 and MSLN-CAR-NK-92 cells. In particular, cancer-friendly overexpressed ECM can hinder the spread of anti-cancer immune cells extravasated from the blood vessels. Another specificity to consider in the TME is the IFP, which is also a tumor-specific phenomenon that applies pressure to tumor blood vessels. In tumors, the rate of cancer cell proliferation is higher than that of angiogenesis. In addition, drainage is not smooth due to defective lymphatic vessels. In this environment, many cancer cells are generated around the blood vessels, and a large amount of ECM around the blood vessels can exert considerable IFP on the blood vessels. An intratumoral situation may arise in which anticancer immune cells are also inevitably affected to some extent by this IFP 40-44. In this study, we verified that MSLN-CAR-NK-92 cells, which showed increased extravasation compared to NK-92 cells, improved the anticancer effect by interacting with more cancer cells. Therefore, if we can quantitatively evaluate the extent to which extravasation has been improved by the CAR design, the anticancer effects of CAR-NK and CAR-T cells on solid tumors can be clearly revealed. To date, there have been no reports on the extent to which the anticancer efficacy of anticancer immune cells varies in solid tumors as a function of extravasation. In this study, we propose, for the first time, a method to quantitatively evaluate the degree of extravasation of anticancer immune cells in tumors through tumor transparency imaging. As a result, the difference in the anticancer effect exerted by MSLN-CAR-NK-92 cells compared to that of NK-92 cells was clearly demonstrated by the distribution and number of pancreatic cancer cell deaths. Quantification of the extravasation of anti-cancer immune cells was possible because tumor transparency imaging can accurately track and display the three-dimensional distribution of anti-cancer immune cells. Unlike the inevitable destruction of tumor blood vessels in tissue sections, tumor transparency imaging allows the imaging of tumor blood vessels in an intact state without blood vessel damage. Therefore, the entire volume occupied by anticancer immune cells inside and outside blood vessels can be accurately determined, and their ratio representing the degree of extravasation can be determined. The extravasation of MSLN-CAR-NK-92 cells was 149% higher than that of NK-92 cells. This result demonstrated the effectiveness of the CAR design in NK cells expressing mesothelin targeting receptor. Through the CAR design of NK cells, the drug permeability and extravasated number of MSLN-CAR-NK-92 cells increased by ~ 57 μm and 3.3 times, respectively, compared to NK cells. Mesothelin scFv, expressed in the ecto-region of CAR of MSLN-CAR-NK-92 cells, has a targeting function in pancreatic cancer cells. It is conceivable that the targeting function mediated by mesothelin scFv may have contributed to the enhanced drug permeability of MSLN-CAR-NK-92 cells in pancreatic tumors compared to NK-92 cells without the MSLN targeting function. In contrast, IL-15 secretion did not appear to influence the migratory behavior of CAR-NK cells *in vivo*, including infiltration and extravasation, within the short observation period of 2 days after cell injection.

Although IL-15 coexpression clearly enhanced NK-92 cell persistence and cytotoxic activity *in vitro*, its *in vivo* effects could not be fully evaluated in the present study. During preliminary *in vivo* tumor growth inhibition experiments, repeated administration of MSLN-CAR-IL-15-NK-92 cells resulted in unexpected mortality of the treated mice after multiple infusions, which prevented reliable assessment of long-term therapeutic efficacy. Although the underlying mechanism was not investigated in this study, previous reports have suggested that constitutive IL-15 expression in engineered CAR-NK cells may lead to excessive NK-cell expansion and systemic inflammatory toxicity *in vivo* 45, 46. These findings suggest that while IL-15 armoring may improve NK-cell persistence, careful regulation of IL-15 expression will likely be necessary to balance enhanced persistence with systemic safety. Further *in vivo* studies will therefore be required to clarify the role of IL-15 in regulating NK-cell persistence, migration, and therapeutic efficacy within the tumor microenvironment.

One limitation of the present study is the use of a subcutaneous tumor model, which does not fully recapitulate the complex tumor microenvironment and anatomical context of native tumors. Subcutaneous models lack organ-specific features such as tissue architecture, vascularization patterns, immune cell composition, and mechanical constraints that may critically influence immune cell trafficking and vascular extravasation *in vivo*. Therefore, although the subcutaneous model provides a convenient and reproducible platform for mechanistic investigation, further validation in orthotopic tumor models will be essential to confirm the generalizability and clinical relevance of our findings. Future studies will focus on evaluating the therapeutic efficacy and immune cell dynamics in orthotopic settings that more closely mimic the physiological tumor microenvironment.

## Conclusion

In this study, the effectiveness of the CAR design of MSLN-CAR-NK-92 cells was demonstrated by enhanced extravasation using tumor transparency imaging. MSLN-CAR-NK-92 cells demonstrated significantly enhanced cytotoxicity and functional activity in *in vitro* tests, including elevated CD107a expression and IFN-γ production, compared to parental NK-92 cells. Moreover, co-expression of IL-15 further improved cell persistence and proliferation, resulting in sustained cytotoxicity over 6 days under cytokine-free conditions. In independent mouse xenograft models, MSLN-CAR-NK-92 cells showed ~2-fold higher CPA50/CPD50 (88 µm - 6887 cells vs. 45 µm - 3509 cells) and ~3-fold higher CDA50/CDD50 (102 µm - 6350 cells vs. 48 µm - 2023 cells) than NK-92 cells, demonstrating superior extravasation and cytotoxicity. In the simultaneously administered xenograft mouse model, the extravasation of MSLN-CAR-NK-92 and NK-92 cells was successfully quantified by calculating the ratio of their total volume inside to that outside the tumor blood vessels. MSLN-CAR-NK-92 cells demonstrated superior extravasation efficiency, with an extravasation fraction of 85.3%, which was approximately 1.5-fold higher than that of NK-92 cells (57.4%) and a 3.3-fold increase in the number of extravasated cells (7,717 vs. 2,311). As a result of improved extravasation, both drug permeability and pancreatic tumor cell death induced by MSLN-CAR-NK-92 cells increased compared to those induced by NK-92 cells.

## Supplementary Material

Supplementary figures.

## Figures and Tables

**Table 1:** Drug permeability and extravasation of NK-92 and MSLN-CAR-NK-92 cells. Data are presented as mean ± SD (n = 3).

Cell type	NK-92 cells	MSLN-CAR-NK-92 cells
Maximum cell penetration depth (CPD_max_) (µm)	89.3 ± 26.6	175.3 ± 10.1
Half of max cell penetration depth (CPD_50_) (µm)	44.7 ± 13.3	87.7 ± 5.0
Cell penetration amount 50 (CPA_50_)	3509 ± 1132	6887 ± 1594
Maximum cell death depth (CDD_max_) (µm)	95.3 ± 33.7	204 ± 33.3
Half of max cell death depth (CDD_50_) (µm)	47.7 ± 16.9	102 ± 16.6
Cell death amount 50 (CDA_50_)	2023 ± 288	6350 ± 1182
No. of extravasated cells	2311 ± 782	7717 ± 200
Volume fraction of extravasation (%)	57.4 ± 8.1	85.3 ± 3.0

## Data Availability

Data will be made available on request.

## Abbreviations

CAR: chimeric antigen receptor
TME: tumor microenvironment
CAR-NK: chimeric antigen receptor-engineered natural killer
PDAC: pancreatic ductal adenocarcinoma
3D: three-dimensional
OE: overexpression
PI: propidium iodide
NSG: NOD-SCID IL2Rγ^null^
DDW: double-distilled water
TUNEL: terminal deoxynucleotidyl transferase dUTP nick end labeling
SEM: standard error of the mean
SD: standard deviation
ANOVA: analysis of variance
CPD50: half of the maximum cell penetration depth
CPA50: cell penetration amount 50
CDD50: half of the maximum cell death depth
CDA50: cell death amount 50
MSLN: mesothelin
CAFs: cancer-associated fibroblasts
