# Supplementary material for: Quantitative assessment of extravasation of IL-15-secreting MSLN-CAR-NK-92 cells using tumor transparency imaging
Source: Theranostics. 2026 Apr 23;16(11):6202–19. doi: 10.7150/thno.125194 (PMC13142241; doi:10.7150/thno.125194)
Supplement: Supplementary file 1 — Supplementary figures. [file thnov16p6202s1.pdf]

Supplementary Information

Quantitative assessment of extravasation of IL-15–secreting MSLN-CAR-NK-92 cells using tumor transparency imaging

Sera Hong[1], Dohyeon Moon[1], Seoin Hwang[2], Mijeong Lee[2], Duck Cho[2, 3,*] and Joon Myong Song[1,*]

[1] College of Pharmacy, Seoul National University, Seoul 08826, Republic of Korea

[2] Department of Health Sciences and Technology, Samsung Advanced Institute for Health Sciences and Technology, Sungkyunkwan University, Seoul 06351, Republic of Korea

[3] Department of Laboratory Medicine and Genetics, Samsung Medical Center, Sungkyunkwan University School of Medicine, Seoul 06351, Republic of Korea

* Corresponding authors: jmsong@snu.ac.kr (J. M. Song), duck.cho@skku.edu (D. Cho)

Contents

Supplementary Figures ········ **3**

Figure S1. ········ **3**
Figure S2. ········ **4**
Figure S3. ········ **5**
Figure S4. ········ **6**
Figure S5. ········ **7**
Figure S6. ········ **8**

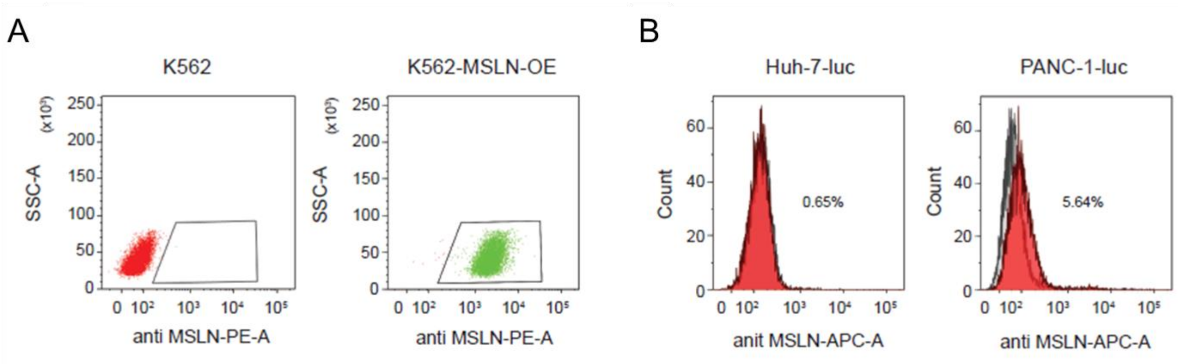

Figure S1. (A) Flow cytometric analysis of transduction efficiency in K562 cells transduced with mesothelin CDS constructs. (B) Flow cytometric analysis of mesothelin expression in Huh-7-luc and PANC-1-luc cells.

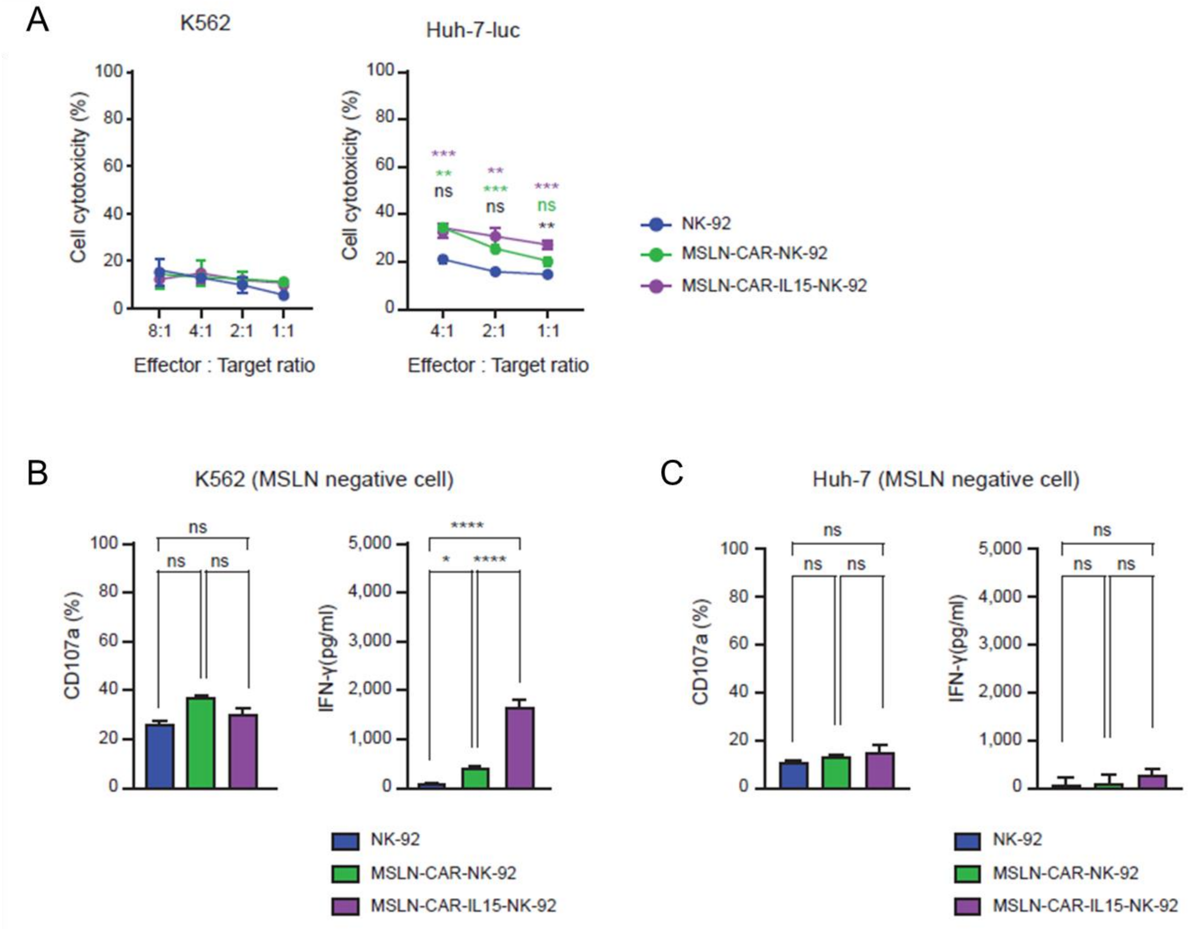

Figure S2. (A) Cytotoxicity of NK-92, MSLN-CAR-NK-92 and MSLN-CAR-IL-15-NK-92 cells against K562 and Huh-7-luc cells at various effector to target ratios. (B) CD107a expression and IFN-γ production by NK-92, MSLN-CAR-NK-92 and MSLN-CAR-IL-15-NK-92 cells after co-culture with K562 and MSLN-OE K562 target cells. (C) CD107a expression and IFN-γ production by NK-92, MSLN-CAR-NK-92 and MSLN-CAR-IL-15-NK-92 cells after co-culture with Huh-7-luc and PANC-1-luc target cells. Data are presented as mean ± standard deviation (SD). Statistical significance was assessed using unpaired t-test (A) and ordinary one-way ANOVA followed by Tukey's multiple comparison test (B, C). * $p < 0.05$, ** $p < 0.01$, *** $p < 0.001$, **** $p < 0.0001$. Colored asterisks indicate statistical comparisons between groups (Green: NK-92 vs MSLN-CAR-NK-92; purple: NK-92 vs MSLN-CAR-IL-15-NK-92; black: MSLN-CAR-NK-92 vs MSLN-CAR-IL-15-NK-92).

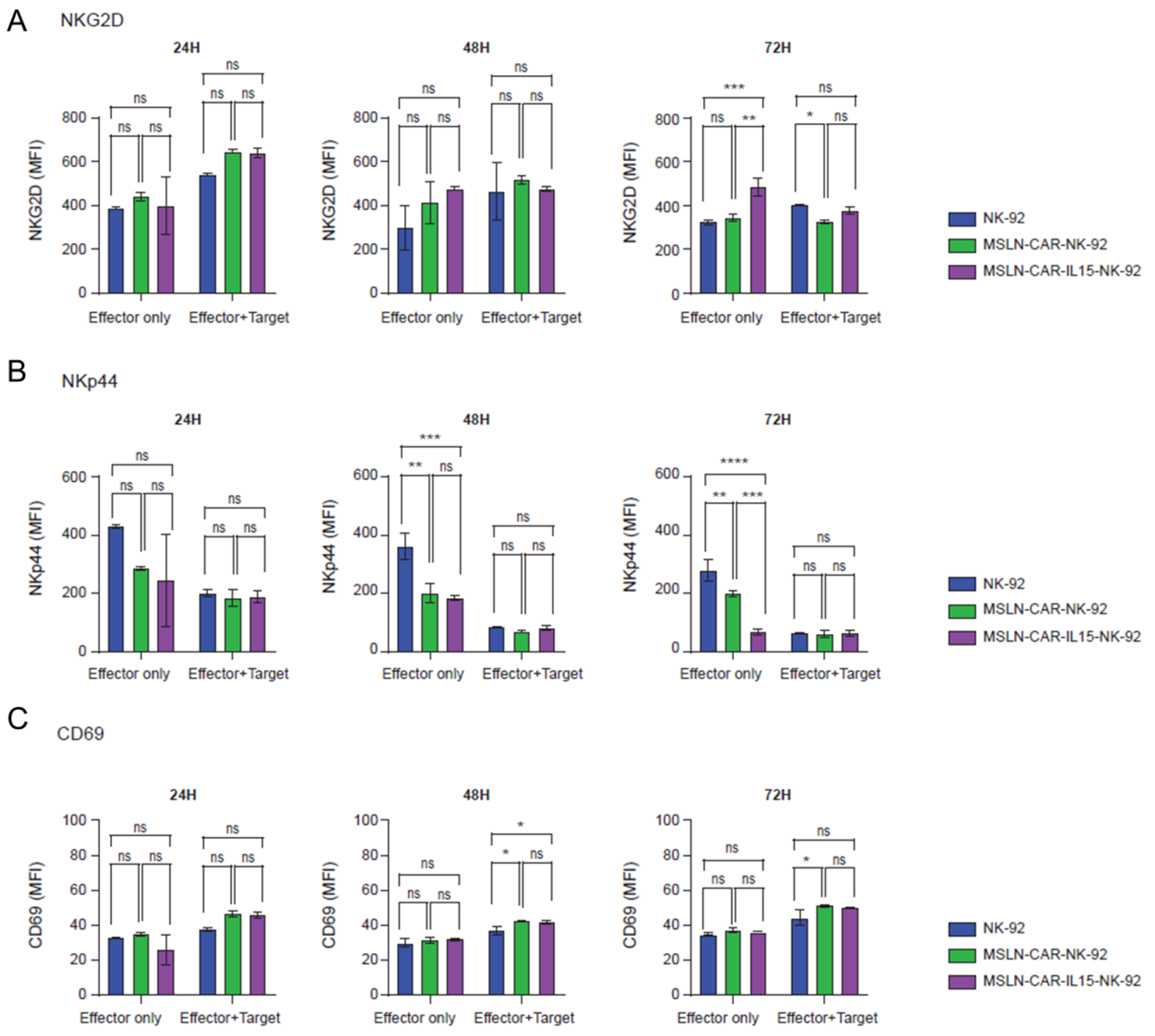

Figure S3. (A-C) Flow cytometric analysis of activation marker expression in NK-92, MSLN-CAR-NK-92, and MSLN-CAR-IL-15-NK-92 cells. Cells were analyzed at 24, 48, and 72 h under effector only condition or after co-culture with target cells. The expression levels of (A) NKG2D, (B) NKp44, and (C) CD69 are presented as mean fluorescence intensity (MFI). Data are presented as mean ± standard deviation (SD). Statistical significance was assessed using ordinary one-way ANOVA followed by Tukey's multiple comparison test * $p < 0.05$, ** $p < 0.01$, *** $p < 0.001$, **** $p < 0.0001$.

A

B

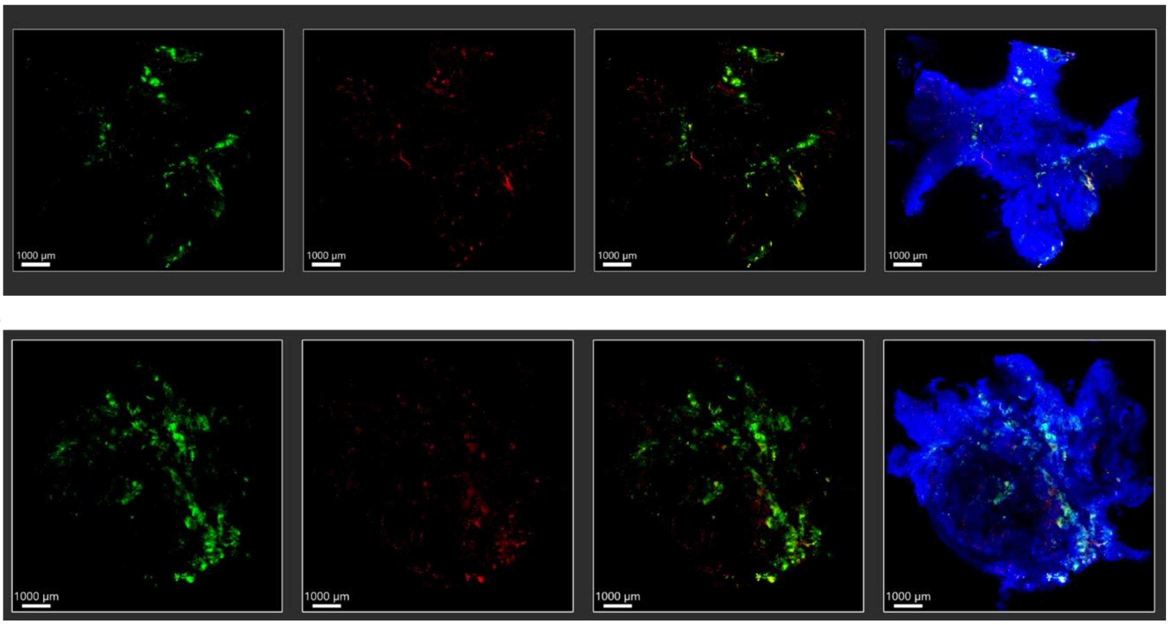

Figure S4. Full cross-sectional confocal mosaic images representing spatial heterogeneity of vascular distribution and immune cell extravasation in PANC-1 pancreatic tumor sections. (A) Tumor injected with NK-92-GFP cells. Green, NK-92 cells (GFP); red, blood vessels; blue, nuclei. (B) Tumor injected with MSLN-CAR-NK-92-GFP cells. Green, MSLN-CAR-NK-92 cells (GFP); red, blood vessels; blue, nuclei. Images were acquired from cleared tumor tissues using confocal microscopy with mosaic tile scanning to capture the entire tumor cross-section. All scale bars, 1000 μm.

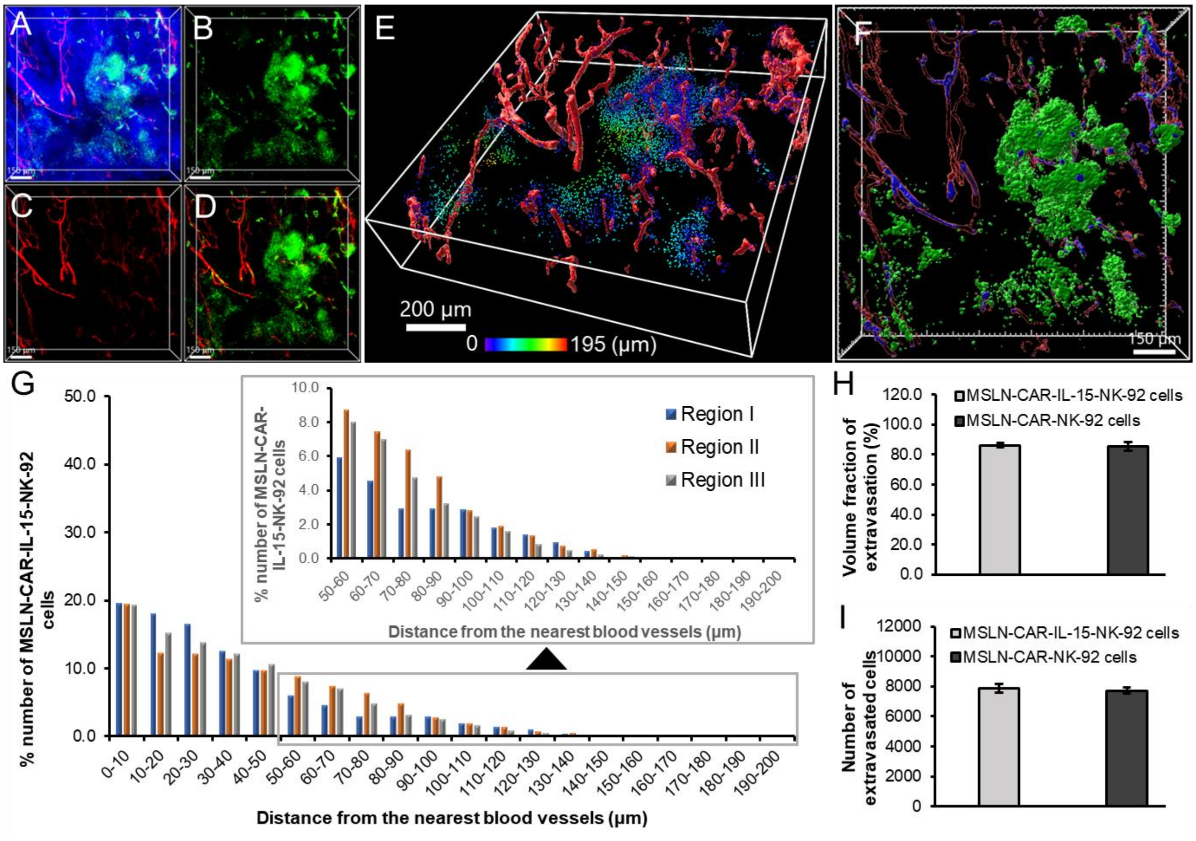

Figure S5. 3D tumor images showing the spatial distribution of (A-F) MSLN-CAR-IL-15-NK-92-GFP cells in pancreatic cancer. (A) Merged image of blood vessels and MSLN-CAR-IL-15-NK-92-GFP cells with nuclei labeled with Hoechst. (B-C) 3D images of (B) MSLN-CAR-IL-15-NK-92 cells (GFP) and (C) blood vessels. (D) Merged image of the MSLN-CAR-IL-15-NK-92 cells and blood vessels. (E) 3D-reconstructed image of (A) (raw image). Spots (MSLN-CAR-IL-15-NK-92 cells) are represented in spectral color based on their distance to the nearest blood vessels. (F) 3D-reconstructed image represents extravasated (green) and intravascular (blue) MSLN-CAR-IL-15-NK-92 cells and vasculature (red). (G) Graphical data signify the percentage number of MSLN-CAR-IL-15-NK-92 cells that were distributed in relation to the distance from the blood vessel. (H) Graph illustrating the extravasation volume fraction of MSLN-CAR-IL-15-NK-92 cells and MSLN-CAR-NK-92 cells. (I) Graph showing the number of extravasated cells of MSLN-CAR-IL-15-NK-92 cells and MSLN-CAR-NK-92 cells. Data are presented as mean ± SD (n = 3). (n = 3 independent biological experiments). Scale bars, (A-D) and (F) 150 μm, (E) 200 μm.

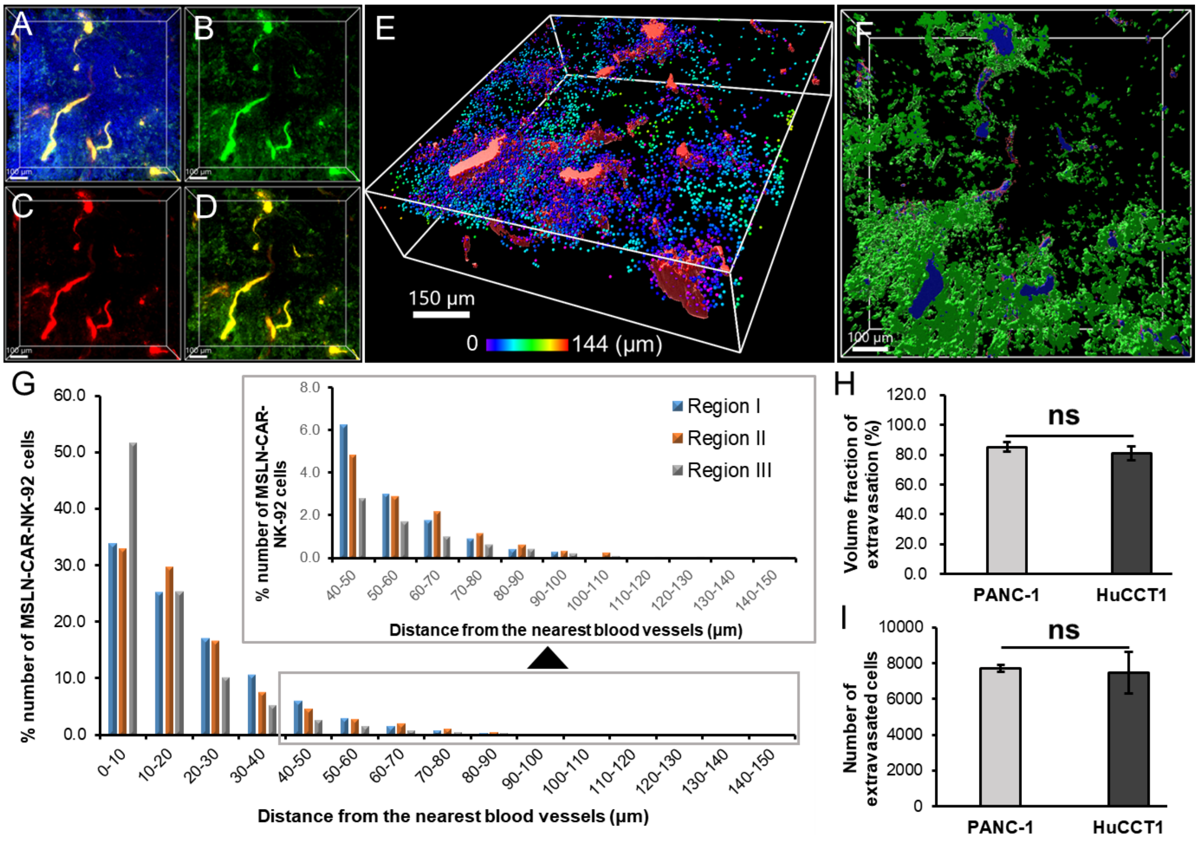

Figure S6. 3D tumor images showing the spatial distribution of (A-F) MSLN-CAR-NK-92-GFP cells in cholangiocarcinoma. (A) Merged image of blood vessels and MSLN-CAR-NK-92 cells with nuclei labeled with Hoechst. (B-C) 3D images of (B) MSLN-CAR-NK-92 cells (GFP) and (C) blood vessels. (D) Merged image of the MSLN-CAR-NK-92 cells and blood vessels. (E) 3D-reconstructed image of (A) (raw image). Spots (MSLN-CAR-NK-92 cells) are represented in spectral color based on their distance to the nearest blood vessels. (F) 3D-reconstructed image represents extravasated (green) and intravascular (blue) MSLN-CAR-NK-92 cells and vasculature (red). (G) Graphical data signify the percentage number of MSLN-CAR-NK-92 cells that were distributed in relation to the distance from the blood vessel. (H) Graph comparing the extravasation volume fraction of MSLN-CAR-NK-92 cells in PANC-1 and HuCCT1 xenograft models. (I) Graph comparing the number of extravasated cells of MSLN-CAR-NK-92 cells in PANC-1 and HuCCT1 xenograft models. Data are presented as mean ± SD (n = 3). (n = 3 independent biological experiments). Scale bars, (A-D) and (F) 100 µm, (E) 150 µm. ns, not significant.
